# Food-Aid Quality Correlates Positively With Diet Quality of Food Pantry Users in the *Leket Israel* Food Bank Collaborative

**DOI:** 10.3389/fnut.2018.00123

**Published:** 2018-12-18

**Authors:** Dana Efrati Philip, Ghada Baransi, Danit R. Shahar, Aron M. Troen

**Affiliations:** ^1^The Nutrition and Brain Health Laboratory, The Institute of Biochemistry Food Science and Nutrition, The Robert H. Smith Faculty of Agriculture, Food and Environment, The Hebrew University of Jerusalem, Rehovot, Israel; ^2^Department of Public Health, Faculty of Health Sciences, The S. Daniel Abraham International Center for Health and Nutrition, Ben-Gurion University of the Negev, Beersheba, Israel

**Keywords:** food insecurity, food bank, food pantry, food aid, nutrition, dietary assessment, survey, food frequency questionnaire

## Abstract

**Introduction:** In many affluent countries, including Israel, networks of food banks and pantries have increasing responsibility to alleviate endemic poverty and food insecurity. While they may relieve acute hunger, their long-term influence on health and well-being is poorly understood.

**Methods:** An exploratory cross-sectional telephone survey assessed both adequacy and quality of food aid provided via food pantries in the *Leket Israel* food bank network, in relation to recipients' dietary needs and health. The quality of food baskets and recipient diets were given a Healthy Portions Score (HPS) to measure compliance with Government guidelines for a “Basic Healthy Food Basket,” and a Nutrient Density Score (NDS) to capture how well the food achieved the recommended dietary allowance (RDA) for vital macro and micronutrients. A total of 105 pantry users were surveyed from 16 pantries around the country.

**Results:** The basket HPS correlated positively and highly significantly with dietary quality (individual NDS) after adjusting for gender, marital status and country of birth (standardized β = 0.22, *p* = 0.03). Nearly half (46%) reported food insecurity with hunger. Two thirds were overweight or obese, and anemia, cardiovascular and metabolic disease were prevalent. The average food basket provides 30% of energy, 55% of protein, 50% of fiber, but only 33% or less of the household requirement for most minerals and vitamins. Only 60% of participants met their estimated energy requirements, and the intake of many essential micronutrients was well below the RDA. Fruits and vegetable portions contributed by *Leket Israel* correlated positively with the dietary quality (individual NDS) after adjustment for the same covariates (Standardized β = 0.20, *p* = 0.04).

**Discussion:** A structured telephone survey proved a feasible method to study the impact of food-aid quality on the nutrition and health of food pantry users in an affluent country. Food baskets with fruits, vegetables and higher quality nutrition were correlated with healthier diets among the recipients. Data correlating food-aid quality and recipient diet and health is essential to effective policy making.

## Introduction

Food provided by food banks and food pantries can be an important source of nourishment for people suffering hunger and food insecurity. Such aid was traditionally a charitable intervention to meet emergent or short-term needs. However, enduring poverty in affluent countries and a confluence of political and societal factors have increasingly driven charitable food banks and pantries to provide food for large segments of society with chronic food insecurity (1–7). The growing reliance on food banks in affluent countries makes it vital to gauge their long-term effect on the health and wellbeing of those who routinely depend on their food.

“Food banks” refers to “wholesale” operations that typically procure and redistribute donated or purchased food through a network of smaller food pantries. These pantries, often local charities that serve different populations and operate independently of one another, have widely varying modes of operation, resources and capacity. As a result, the quality and quantity of food reaching aid-recipients varies widely. Moreover, because distributing perishable nutrient-dense food (i.e., meat, dairy, fruits, and vegetables) requires considerable investments in infrastructure and expertise, many food banks distribute staples that do not require refrigeration, have a long shelf-life and are replete in calories but poor in essential micronutrients and minerals (7–9). Habitual consumption of such poor quality food might compound the ill effects of poverty-driven high-calorie and low-nutrient diets (10–12) associated with obesity, mineral, and vitamin deficiencies, and a high risk of chronic diet-related diseases including high blood pressure, diabetes, cardiovascular disease, and anemia (13–17). Food banks that provide nutrient-dense perishable food such as fruit and vegetables are likely to improve the quality of the food distributed by their partners and importantly also the nutrition and health of the households they serve. Nevertheless, because the impact of “wholesale” foodbanks is indirect, it is essential to develop the means to ascertain the predicted benefit to the individual recipient or household and its magnitude.

The difficulty of assessing both the nutritional quality of food pantry parcels and the individual aid-recipient's nutrition and health may explain why such data are sparse (18, 19). Two extensive reviews of the scientific literature published in 2017 found only 9 studies of pantry food quality and 16 studies of user diet quality (conducted in the USA, Canada, Australia, and France) (20, 21). Importantly, these reviews conclude that the dietary intake of pantry users failed to achieve dietary recommendations and that food pantries were largely unable to support healthy diets. They suggest increasing distribution of nutritious perishable food such as fruit and vegetables to improve the quality of pantry users' diets. However, no study has yet quantified the health benefit to aid recipients of fruit and vegetable distribution by wholesale food banks through food pantry networks.

Israel is a prominent example of an affluent country with widespread and persistent food insecurity. Government data indicate that nearly one in five Israelis (18.9%) experience some degree of food insecurity, while 8.6% suffer severe food-insecurity. This means that 747,000 people including 285,000 children in 243,000 households are severely food insecure (22). An estimated 1,200 non-profit organizations and charities, the majority of which are small local charities and food pantries and a handful of larger national organizations, have emerged to meet this crying need. A recent government pilot “National Food Security Initiative” aims to distribute a modest monthly allowance of fruits and vegetables, staple foods, and cash benefits equivalent to 250 NIS (~65 US dollars) per month to 10,800 families throughout the country (23). To avoid creating new entitlements and bureaucracy the pilot is conceived as a sort of “public-private partnership” that relies on the collective resources, infrastructure, reach, and expertise of three philanthropic food banks, *Leket Israel, Latet, and Eshel Jerusalem*.

*Leket Israel* is the largest food bank in Israel. A “wholesale” operation, it specializes in collecting and redistributing highly perishable foods and rescuing surplus fruits and vegetables (9). It currently provides food to ~220 independent local food pantries, soup kitchens and other non-profit organizations (NPOs) throughout the country with no direct contact with the final aid recipients. The aid from Leket Israel (largely fruits and vegetables) is combined with other food that each NPO procures and distributes according to their own criteria. As a result, clients of different food pantries receive food aid of differing quantity and quality.

We designed this exploratory study of adult Israeli food pantry users to examine the relationship between the quality of the food baskets distributed by multiple NPOs and the recipients' nutrition and health. Our study had three specific aims: to develop a feasible method of evaluating the nutritional quality of both the food supplied and the diets of the food-insecure population that regularly receives aid from *Leket Israel*'s food security network but has no direct contact with the food bank; to measure the contribution of the *Leket Israel* food bank to the nutritional value of the food baskets distributed by their partner food pantries; and to gauge how the quality of the food baskets provided to households by different food pantries affects the food security, habitual dietary quality, and health of individual aid recipients in those households.

## Methods

The Hebrew University Institutional Ethics Committee for Research Involving Human Subjects granted ethical approval for this study.

The study was designed as a structured, cross-sectional telephone survey of food-security, diet and health, among 100 food-aid recipients receiving assistance from non-governmental/non-profit food pantries (NPOs) partnering with the “Leket Israel” food bank. At the time the study was designed, there were ~180 NPOs serving 140,000 clients throughout the country that partnered with Leket Israel and included their fruits and vegetables in their food baskets.

We classified the NPOs and selected those invited to participate in the study based on their location, size, method of food distribution, and demographic characteristics of their clientele. The obtained convenience sample of pantries represented all regions, sizes, methods of operation, as well as characteristics such as the foods distributed and their sources, Leket Israel's contribution to the food baskets, and the NPO's clientele demographic. We aimed to survey between 5 and 10 individual clients from each NPO. If a selected NPO declined to participate, an alternate NPO with similar characteristics was invited. Ultimately, 16 of 22 invited NPOs enlisted in the survey with a total of 105 participants (Table 1).

**Table 1:** Characteristics of the food pantries participating in this study.

**Pantry number**	**Main population served**	**Geographical area**	**Number of households served by the pantry**	**Type of food distributed**	**Number of individual aid-recipients recruited for the study**
1	Jewish mixed	North	80	FV	7
2	Jewish mixed	North	95	FV, DF, D	7
3	Jewish elderly	South	50	FV	8
4	Druze	North	120	FV, DF	9
5	Bedouin	South	1500	FV, FD, D, M	4
6	Muslim Arab	Center	600	FV, FD, Eggs, M	7
7	Jewish mixed	Center	560	FV, DF, M	2
8	Jewish mixed	Center	150	FV, DF, D, Eggs, M	7
9	Jewish mixed	North	380	FV, DF, D, M	7
10	Jewish mixed	Center	275	FV, DF	3
11	Jewish mixed	North	1000	FV, DF, D, Eggs, M	4
12	Jewish mixed	North	525	FV, DF, M	13
13	Jewish mixed	North	300	FV, DF, D	10
14	Jewish mixed	North	400	FV, DF, D, M	11
15	Jewish mixed	South	170	FV, DF	1
16	Jewish mixed	South	380	FV, DF, D, M	5

*The food pantry staff provided food pantry descriptions. Food pantries serving “mixed population” serve anyone in need rather than particular sectors and therefore include elderly individuals, large households, single mother households, new immigrants, etc. FV, fruits and vegetables; DF, dry foods; D, dairy; M, meat*.

Participating NPO staff recruited volunteers from among their clients so as to obtain a representative demographic of the NPO's clientele. Depending on the NPO's normal modus operandi NPO staff invited all clients to participate in the survey at the client's home, at the food distribution point, or by phone. The staff explained the study to participants and obtained signed, written, informed consent from those who volunteered to participate. Written informed consent explicitly specified that participation was completely voluntary, that participants had the right to decline to answer any questions or to withdraw their consent at any time, and that a choice to decline would in no way impinge on the assistance received from the NPO. Upon consent, the phone number and first name of the participant were provided to the survey interviewer. In one NPO, subjects were recruited indirectly through flyers inserted in the food baskets rather than by NPO's staff. In this case, clients who wished to participate in the study contacted the researchers directly by phone and sent the informed consent form electronically. Individuals were excluded if they were unable to understand the questions or any of the interview languages, suffered from any physical or mental disability that might inhibit comprehension, or were unable or unwilling to sign the written informed consent.

Participants answered a structured telephone interview that consisted of an explanation regarding the study; a demographic questionnaire; a food security questionnaire; self-reported anthropometric measurements and health status; and a food frequency questionnaire (FFQ) (see Online Supporting Information). Before beginning the survey, the telephone surveyor obtained verbal confirmation of the written informed consent, clarifying once more that participation in the survey was completely voluntary, that participants had the right to decline to answer any questions or to withdraw their consent at any time, and that a choice to decline would in no way impinge on the assistance received from the NPO. Subjects completing the survey were compensated for their time with supermarket food vouchers valued at 100 NIS (around 25 US dollars). This modest amount was deemed by the researchers and ethics committee to be an appropriate incentive and compensation for participants' time without being coercive.

Household food security was measured using the United State Department of Agriculture 18-item Household Food Security Survey Module (24), adapted for use in Israel by the National Insurance Institute of Israel (*Hamosad l'Bituah Leumi*) (22). Missing data were imputed according to USDA guidelines (24). Food security scores were recorded as the number of positive answers (divided by the number of possible answers−18 for households with children or 10 for families without children under the age of 18). Households were classified according to their score as either food secure, food insecure without hunger, food insecure with moderate hunger, or food insecure with severe hunger.

Participants were asked to report past or current diagnoses of non-communicable, diet-related medical conditions—anemia, osteoporosis, high cholesterol, high blood pressure, high triglycerides, stroke, and cancer. Participants also rated their general health as “very good, good, not so good, or not good at all.” Body Mass Index (BMI) was derived from self-reported height and weight according to the formula: BMI = weight [Kg]/(height [m])^2^. Participants were classified as having normal or unhealthy weight according to the standard categories: BMI < 18.5–Underweight; 18.5>BMI < 25–Normal; 25>BMI < 30–Overweight; BMI>30–Obese. These items were adopted from the Israeli National Health and Nutrition Survey (25).

Individual dietary intake was assessed using an Israeli semi-qualitative Food Frequency Questionnaire (FFQ) that was originally designed to be administered in person (26). For telephone administration, we provided participants with an illustrated food portion guide by e-mail or by NPO staff prior to the survey. The questionnaire consists of 126 items that are typically consumed in the Israeli diet. Participants were asked how frequently and how much they typically ate of each food item during the preceding year. Daily energy, protein, fiber, vitamin and mineral, and fruit and vegetable intakes were calculated, allowing for comparison of these data to age and sex-specific dietary requirements. By convention, implausible FFQ responses were excluded from the analyses where self-reported energy intake fell below 600 or above 4,000 kcal/d (27).

Participants were also asked about their knowledge and attitudes regarding nutrition, and whether they would be willing hypothetically to participate in future research and provide access to their medical records. When participants volunteered relevant information beyond the questionnaire, it was recorded as qualitative data. Interviews were conducted in Hebrew and Arabic, using translated questionnaires.

In addition to referring participants to the survey, NPO staff also recorded the contents of a food basket that the participating household received and responded to a questionnaire regarding their operations, food procurement and distribution, and to their knowledge and interest in nutrition. Food basket and participant survey data were collected on routine distribution days that did not precede any holiday.

We devised two quality scores to evaluate the nutrient content of both the individual diets and food baskets (for a more detailed account see the Online Supporting Information):

1. *The Healthy Portions Score (HPS)* measures adherence to the “Basic Healthy Food Basket Guidelines” established by the Government of Israel's National Nutritional Security Council and Ministry of Health (28). These guidelines define adequate portion sizes for each of 5 healthy food groups (whole grains, fruits, vegetables, protein-rich foods, and fats and oils) and the number of recommended daily portions for individual consumption according to age and sex. The HPS is a food-based score rather than a nutrient-based score. The division of total healthy portions by total energy is meant to account for unhealthy foods in the diet such that the HPS score increases as the number of healthy portions in the diet increases, but decreases with increasing total energy intake. Because the score is driven by the *absolute* number of healthy portions, the higher the score the higher the quality of food or diet.
2. *The Nutrient Density Score (NDS*) captures the degree to which the diet achieves the recommended dietary allowance (RDA) for vital macro and micronutrients, divided by the overall energy density of the food. Because the NDS assigns a single number score to express the overall density of multiple nutrients in foods, it can be used as a simple index to compare the *relative* nutritional quality of different foods (29, 30). In a similar way, it can be calculated for whole diets, as we did here. In this case it expresses the diet's ability to supply the RDA for the nutrients included in the score, assuming that energy intake is adequate. A score of 1 indicates a food or diet that provides 100% of the RDA at the required energy intake; a score < 1 indicates some degree of deficiency, and a score higher than 1 indicates possible excess.

### Statistical Analysis

Analyses were performed using SPSS version 24.0 (IBM, Armonk, NY). Distribution measures (center and spread—Median, Mean, SD, quartile ranges) were computed for continuous variables and frequency tables were generated for discrete variables. Main outcomes included dietary quality (FFQ energy, protein, fiber and micronutrient intake, fruit and vegetables consumption, and derived HPS and NDS scores), self-reported health and food security status. Univariate analysis was used to identify simple associations and potential covariates, using bivariate correlations, *t*-tests or one-way ANOVA for continuous variables and the χ^2^ test for discrete variables as appropriate. Fixed factors included food basket total energy, protein, fiber, micronutrient, fruit and vegetables, and basket quality scores (bHPS and bNDS) as well as Leket Israel's contribution to these factors. All demographic factors, nutritional views and habits, such as smoking, and survey data were examined for collinearity with primary outcomes as covariates. Factors found in univariate analysis to show significant associations or trends for association (*P* < 0.1) were entered into the multivariable model and the most parsimonious models were selected in the final analysis. Unless specified otherwise, a *P*-value lower than α = 0.05 was considered statistically significant.

## Results

### Study Flow and Feasibility

**Figure 1:**
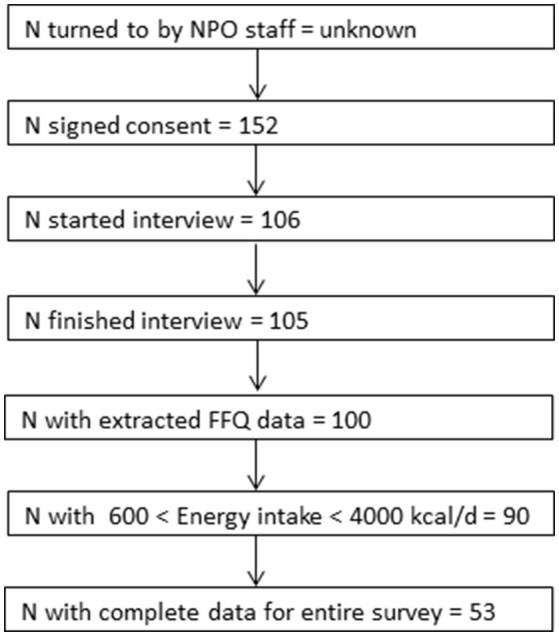
Study population flow chart.

Twenty-two food pantries/NPOs were invited to participate in the study and 16 agreed to participate. Their characteristics are given in Table 1. Half were based in the Northern region (8 of 16) accounting for nearly two thirds of participants in this study. The study flow is depicted in Figure 1. A total of 152 clients consented to participate in the study and 106 were sequentially interviewed to ensure that the enrollment target was met. Of these, 105 successfully completed the phone interview. Only one participant failed to complete an interview after beginning it. The mean interview duration was 58 ±18 min (mean± SD). Twenty-two interviews were done in Arabic and the rest in Hebrew. 73.3% of the clients agreed theoretically to provide access to medical records in the future.

### Population Characteristics

Table 2 gives the population characteristics. The mean age was 51.1 ± 14.5 years (median age 48) and 77.1% were female. The majority of those surveyed identified as Israeli Jews, with the remainder either Israeli Muslims, Druze, Christian or Others (75.2, 12.4, 8.6, 1.9, and 1.9%, respectively). About a third of the population earned a salary during the 3 months prior to the interview, 40% received a disability allowance or pension, and 25.7% were unemployed, either by choice or not. Mean income reported from all sources (including benefits) was 4,458 ± 2,497 NIS (Median 4000 NIS, when 1 NIS = ~0.285 US $) and the mean number of household members was 4.4 ± 2.5 (Median 4). 64.8% of households included children under the age of 18. The vast majority of participants (96%) reported some degree of interest in the relationship between nutrition and health.

**Table 2:** Characteristics of the aid-recipients participating in the study.

	**Mean ± SD**	**Median**
Age	51.1 ± 14.5	48
Income, NIS (*N* = 96)	4,458 ± 2,497	4,000
Number of household members	4.4 ± 2.5	4
	**N**	**(%)**
Children < 18 y old in household (yes)	68	(64.8)
Gender (female)	81	(77.1)
**INTEREST IN NUTRITION AND HEALTH (*****N*** **=** **101)**		
To a very large extent	43	(42.6)
To a large extent	38	(37.6)
To a small extent	16	(15.8)
Not at all	4	(4.0)
**ETHNICITY**		
Jewish	79	(75.2)
Muslim Arab	13	(12.4)
Christian Arab	2	(1.9)
Druze	9	(8.6)
Other	2	(1.9)
**MARITAL STATUS**		
Unmarried	4	(3.8)
Married	59	(56.2)
Divorced/separated	31	(29.5)
Widowed	11	(10.5)
**EMPLOYMENT DURING THE LAST 3 MONTHS**		
Disability	24	(22.9)
Salaried/self-employed	36	(34.3)
Pensioner	18	(17.1)
Unemployed	13	(12.4)
Housewife	14	(13.3)
Smoking (yes, currently)	25	(23.8)

*All data are self-reported. Total sample size - 105 unless stated otherwise*.

*NIS, New Israeli Shekel*.

### Self-Reported Food Security and Health

Table 3 describes the food security and health status of the study population. Although it included only food pantry users, 17.1% reported a food secure household. 37.1% of participants reported food insecure households but not experiencing hunger, while 45.7% suffering from food insecurity with moderate or severe hunger. When asked to describe their subjective feeling of health, the majority of the population (57.1%) reported their health as “good” or “very good,” while 27.6% said it was “not so good” and 15.2% responded their health was “not good at all.” Over a third of the population reported having been diagnosed or presently suffering from conditions related to metabolic syndrome or cardiovascular disease such as high blood pressure or high cholesterol (40 and 33.3%, respectively) and 22.9% reported a history of high triglycerides. 10.5% of the participants reported having a diagnosis of diabetes. When asked about non-communicable diseases involving nutritional deficiencies, 36.2% reported a previous diagnosis of iron-deficiency anemia, and 11.4% of osteoporosis. Based on self-reported height and weight, mean body mass index (BMI) was 28 (Median 26.9, SD ±6.5). More than a third of the study population were classified as obese (34.3%), and 22.9% were over-weight. Only 35.2% had normal BMI and only 3 participants were underweight. The true prevalence of unhealthy weight in this study is likely to be somewhat higher than these values, because people typically understate their weight and overstate their height compared to measured data.

**Table 3:** Food security, health, and anthropometric status of the study participants.

	***N***	**%**
**FOOD SECURITY**		
Food secure	18	17.1
Food insecure without hunger	39	37.1
Food insecure with moderate hunger	40	38.1
Food insecure with severe hunger	8	7.6
**GENERAL HEALTH**		
Very good	16	15.2
Good	44	41.9
Not so good	29	27.6
Not good at all	16	15.2
**NON COMMUNICABLE DISEASES (NCDs)**		
High blood pressure	42	40.0
Anemia	38	36.2
High cholesterol	35	33.3
Triglycerides	24	22.9
Osteoporosis	12	11.4
Diabetes	11	10.5
Cancer	6	5.7
Stroke	1	1.0
**BODY MASS INDEX (BMI)(*****N*** **=** **100)**		
Underweight (< 18.5)	3	2.9
Normal weight (18.5-24.9)	37	35.2
Over-weight (25-29.9)	24	22.9
Obese (>30)	36	34.3
	**Mean BMI** **±** **SD**	**Median BMI**
	28 ± 6.5	26.9

*All data are self-reported. Total sample size = 105 except where indicated otherwise. NCDs, Non-communicable diseases; BMI, Body mass index*.

### Pantry Users' Dietary Quality

**Figure 2:**
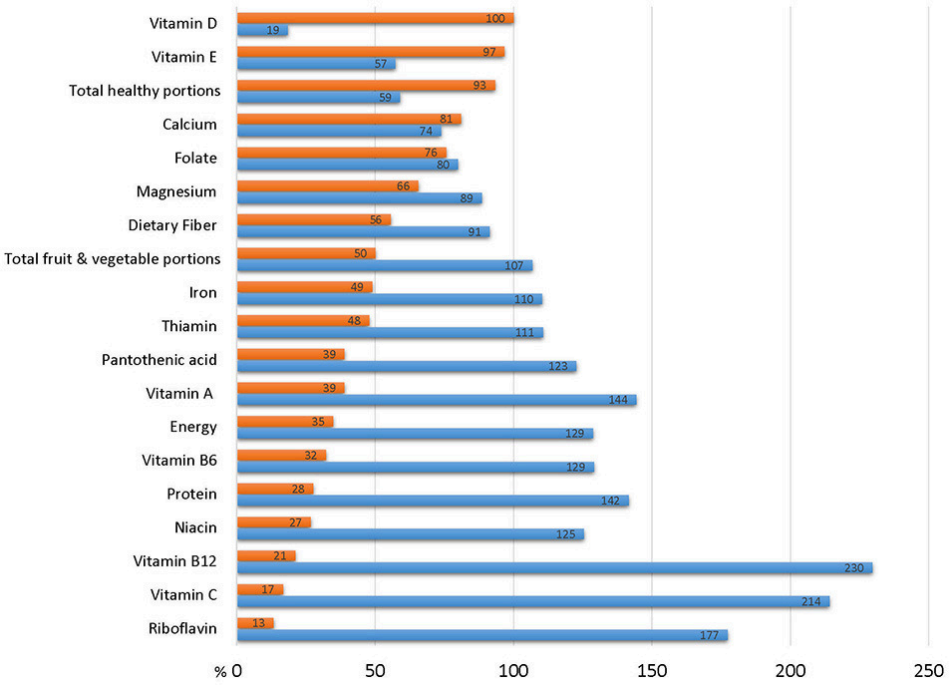
Dietary intake of study participants in in relation to recommended daily allowance (RDA). Blue bar represent mean dietary intake of the study cohort expressed as % of RDA. Red bars represent % of survey participants whose habitual diet fails to achieve the RDA.

**Figure 3:**
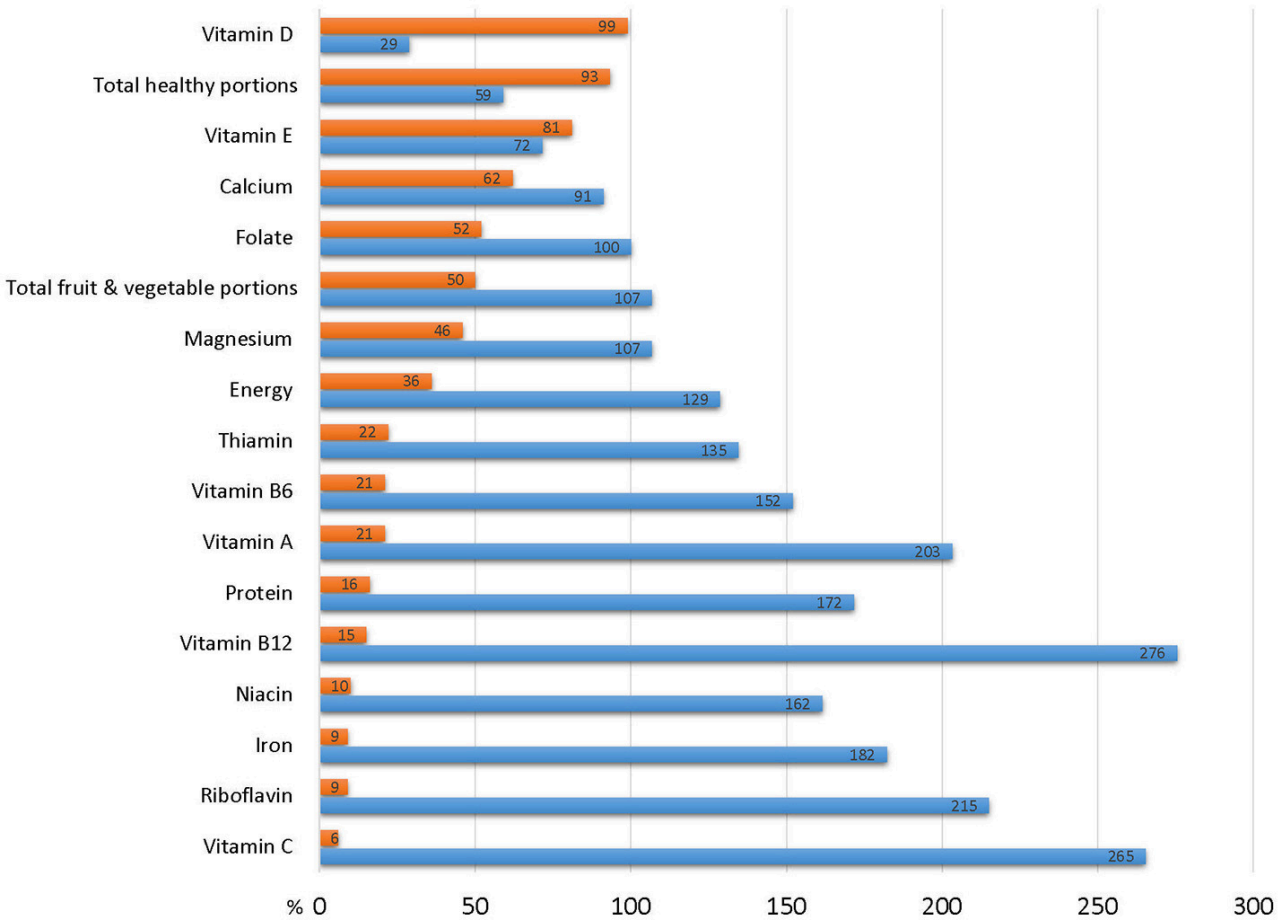
Dietary intake of study participants in in relation to estimated average requirement (EAR). Blue bar represent mean dietary intake of the study cohort expressed as % of EAR. Red bars represent % of survey participants whose habitual diet fails to achieve the EAR.

Table 4 describes the self-reported dietary intake for the study population as measured by FFQ. Only 4.4% consumed enough healthy portions per day to meet the government's “Basic Healthy Food Basket Guidelines,” and only half of the population met the recommended intake of fruit and vegetables. The mean energy intake was 1,974 ± 661 kcal/d (Mean ± SD, Median 1,886 kcal) which represents 128.7% of the mean recommended intake. Nevertheless, only 64% of participants reported intake that met their estimated energy requirements (EER). The situation was better for protein, with mean consumption of 80.9 gr protein per day, and over 72% reaching recommendations. However, the population's intake of many essential micronutrients was well below the recommended daily allowance (RDA). For example, only half of the population achieved the RDA for thiamine (52.2%) and iron (51.1%), less than half consumed enough fiber (44.4%), magnesium (34.4%), folate (24.4%), calcium (18.9%), vitamin E (3.3%), and none (!) reached the recommendation for vitamin D. Figure 2 depicts the high proportion of subjects not reaching the RDA for these nutrients, and Figure 3 shows how, in addition the proportion of the study population failing to achieve the Estimated Average Requirements (EAR). These data are characteristic of poor quality of diet: some of the population consume excess calories, others fail to meet their energy needs, but the majority are deficient in essential micronutrients.

**Table 4:** Quality of habitual individual dietary intake as assessed by Food Frequency Questionnaire (FFQ).

**Nutrient**	**Mean ± SD**	**Dietary Requirement (EER or DRI)**	**Mean intake (% recommended)**	**% participants achieving recommended intake**
Total healthy portions	14.2 ± 5.7	24.1	59.0	4.4
Total fruit & vegetable portions	7.6 ± 4.1	7.1	106.8	50.0
Individual Healthy Portion Score (iHPS—portions /1000 kcal)	7.4 ± 2.0	n.a.	n.a.	n.a.
Individual Nutrient Density Score (iNDS—%RDA/100 kcal)	6.2 ± 1.7	n.a.	n.a.	n.a.
Energy (kcal)	*1, 974.5*±660.9	1640.5	128.7	64.0
Protein (gr)	80.9 ± 30.3	60.2	141.6	72.4
Dietary Fiber (gr)	23.0 ± 8.1	25.2	91.4	44.4
Calcium (mg)	779.8 ± 403.6	1056.6	73.8	18.9
Iron (mg)	11.5 ± 3.8	10.4	110.3	51.1
Magnesium (mg)	303.5 ± 104.9	342.9	88.5	34.4
Vitamin A RAE (mcg)	*1, 075.6*±757.7	745.4	144.3	61.1
Vitamin E (mg)	8.6 ± 3.3	15.0	57.3	3.3
Vitamin D (IU)	115.0 ± 85.2	621.6	18.5	0.0
Vitamin C (mg)	167.5 ± 103.5	78.2	214.1	83.3
Thiamin (mg)	1.2 ± 0.5	1.1	110.6	52.2
Riboflavin (mg)	2.0 ± 0.9	1.1	177.3	86.7
Niacin (mg)	18.2 ± 6.3	14.5	125.4	73.3
Pantothenic acid (mg)	6.1 ± 2.3	5.0	122.7	61.1
Vitamin B6 (mg)	1.8 ± 0.7	1.4	129.0	67.8
Folate (mcg)	319.6 ± 117.6	400.0	79.9	24.4
Vitamin B12 (mcg)	5.5 ± 4.8	2.4	229.6	78.9

*N = 90. Individuals reporting energy values below 600 kcal/day or greater than 4000 kcal/day were excluded. Estimated energy requirement (EER) was calculated for each individual according to the Mifflin-Jeor formula, assuming sedentary lifestyle as default activity level. Required vitamin and mineral intake was set for each participant according to age and sex specific USA Recommended Daily Allowances (RDA) or Adequate Intakes (AI) for each nutrient and healthy portions requirements recommended by the National Nutrition Security Council. n.a. Not applicable*.

### Food Basket Quality

**Figure 4:**
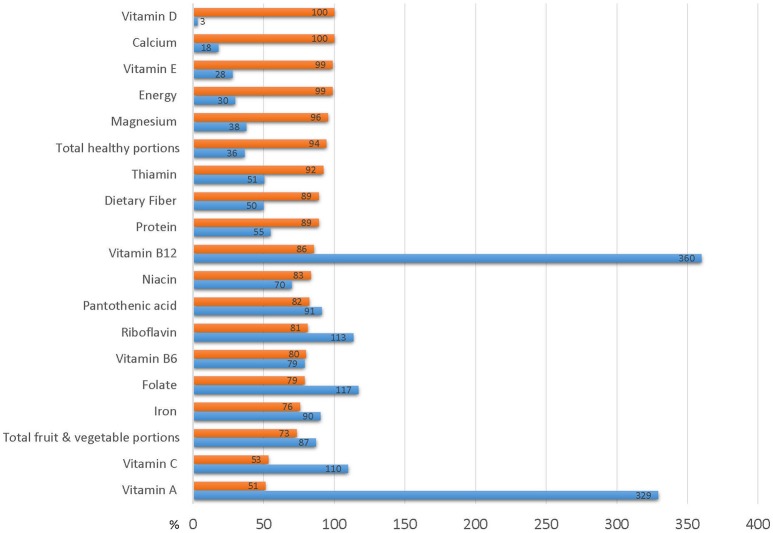
Basket contribution to household dietary requirements Blue bars represent % of household requirement provided by average food aid basket. Red bars represent % of baskets that do not meet household requirements.

Table 5 describes the quality, nutrient content and adequacy of food baskets in relation to household nutritional requirements, assessed by the number of household members, their age and gender. On average, food baskets provided approximately one third of the recommended number of healthy portions required per household (36%) but almost all of the recommended portions of fruits and vegetables (87%). The average basket provided only 29.8% of a household's required energy, 54.9% of their protein and 49.9% of their recommended allowance of fiber. However, the distribution of basket content of most nutrients was skewed to the left. Only 1.1% of the baskets provided the total household energy requirements, and only 11.1% met the requirements for protein and dietary fiber. Analysis of the basket micronutrient content revealed their limited dietary quality. Less than one third of the baskets provided the full household requirement for most minerals and vitamins, only 14.4% of baskets provided the recommended total number of healthy portions, and only one quarter of the baskets supplied the number of fruit and vegetable portions recommended per household (Figure 4).

**Table 5:** The quality, nutrient content and adequacy of food baskets in relation to household nutritional requirements.

**Nutrient**	**Mean**	**SD**	**Portion of household requirement provided on average by baskets (%)**	**% of baskets to provide household requirement**
Total healthy portions	191.4	136.9	36.4	14.4
Total fruit & vegetable portions	136.9	110.6	87.0	25.6
Basket HPS (portions/1000 kcal)	20.0	20.3	Not applicable	Not applicable
Basket NDS (%RDA/100 kcal)	0.3	0.3	Not applicable	Not applicable
Energy (kcal)	16,641.9	12,749.3	29.8	1.1
Protein (gr)	556.8	483.6	54.9	11.1
Dietary Fiber (gr)	313.8	239.5	49.9	11.1
Calcium (mg)	4,557.5	3761.6	17.9	0
Iron (mg)	167.5	159.1	90.1	24.4
Magnesium (mg)	3,066.4	4,492.9	37.7	4.4
Vitamin A RAE (mg)	42.3	58.8	329.4	48.9
Vitamin E (mg)	93.4	71.9	27.9	1.1
Vitamin D (IU)	15.5	28.2	3.2	0
Vitamin C (mg)	1,743.0	1,424.6	109.8	46.7
Thiamin (mg)	11.9	7.8	50.5	7.8
Riboflavin (mg)	24.4	33.8	113.4	18.9
Niacin (mg)	214.0	253.6	69.9	16.7
Pantothenic acid (mg)	81.4	120.6	91.2	17.8
Vitamin B6 (mg)	22.2	18.5	79.0	20.0
Folate (mcg)	7,725.3	9,647.6	117.1	21.1
Vitamin B12 (mcg)	121.0	278.1	360.0	14.4

*N = 90. Basket quality was calculated based on the food content of a routine basket provided to each household surveyed. The amount of food provided per week was calculated and compared with the weekly household requirements taking into account the age and gender of all members of the study participant's household. Households of participants reporting energy intake values below 600 kcal/day or greater than 4000 kcal/day were excluded from the analysis. Dietary requirements are based on the USA Recommended Daily Allowance (RDA) for nutrients and by the household energy and healthy portions requirements recommended by the National Nutrition Security Council*.

Table 6 gives the contribution of food provided by the Leket Israel food bank to the nutrient content of the food pantries' food-aid baskets. The food provided by Leket Israel makes up 64.7% of the total number of healthy portions and 89.5% of the fruit and vegetable portions in an average food pantry or NPO basket. Although Leket Israel contributes 15.9% of the baskets' total energy content and 16.2% of the protein, Leket Israel's contribution makes up half of the fiber, 67.9% of the vitamin C, 55.4% of the calcium, 41.5% of the vitamin A and between 17 and 36% of the content of vitamin B1, B5, and B6, vitamin E, Folate, and Magnesium. These findings indicate the very substantial contribution of fruit and vegetables to the overall nutritional quality and nutrient-density of food-aid parcels.

**Table 6:** The contribution of food provided by the Leket Israel food bank to the nutrient content of the food pantries' aid baskets.

**Nutrient**	**Total weighted mean nutrient content**	**NPOs portion of the total**	**Leket Israel's portion of the total**	**Leket Israel % of total**
Total Healthy portions	191.4	67.5	123.9	64.7
Total Fruit & vegetable portions	136.9	14.4	122.5	89.5
Energy (kcal)	16,641.9	14,004.3	2,637.6	15.9
Protein (gr)	556.8	466.4	90.4	16.2
Dietary fiber (gr)	313.8	156.9	156.9	50.0
Pantothenic Acid (B5, mg)	81.4	67.4	14.0	17.2
Calcium (mg)	4,557.5	2,033.7	2,523.8	55.4
Iron (mg)	167.5	128.3	39.2	23.4
Magnesium (mg)	3,066.4	1,979.6	1,086.8	35.4
Thiamin (B1, mg)	11.9	8.1	3.8	31.9
Riboflavin (B2, mg)	24.4	20.9	3.5	14.3
Niacin (B3, mg)	214.0	175.0	39	18.2
Vitamin A RAE (mg)	42.3	24.8	17.5	41.5
Vitamin E (mg)	93.4	66.7	26.7	28.6
Vitamin C (mg)	1,743.0	560.0	1,183.0	67.9
Vitamin D (IU)	15.5	14.0	1.5	9.7
Vitamin B6 (mg)	22.2	14.2	8.0	36.0
Folate (mcg)	7,725.3	5,664.7	2,060.6	26.7
Vitamin B12 (mcg)	121.0	120.5	0.5	0.4

*Values are weighted means: the contribution of each NPO's routine basket to the mean was weighted by the number of individual aid-recipients recruited for the study from that NPO out of the total sample size of 90 participants with valid dietary data (see Table 1)*.

### Association Between Food Basket Quality, Individual Diet Quality Food Security and Health

**Figure 5:**
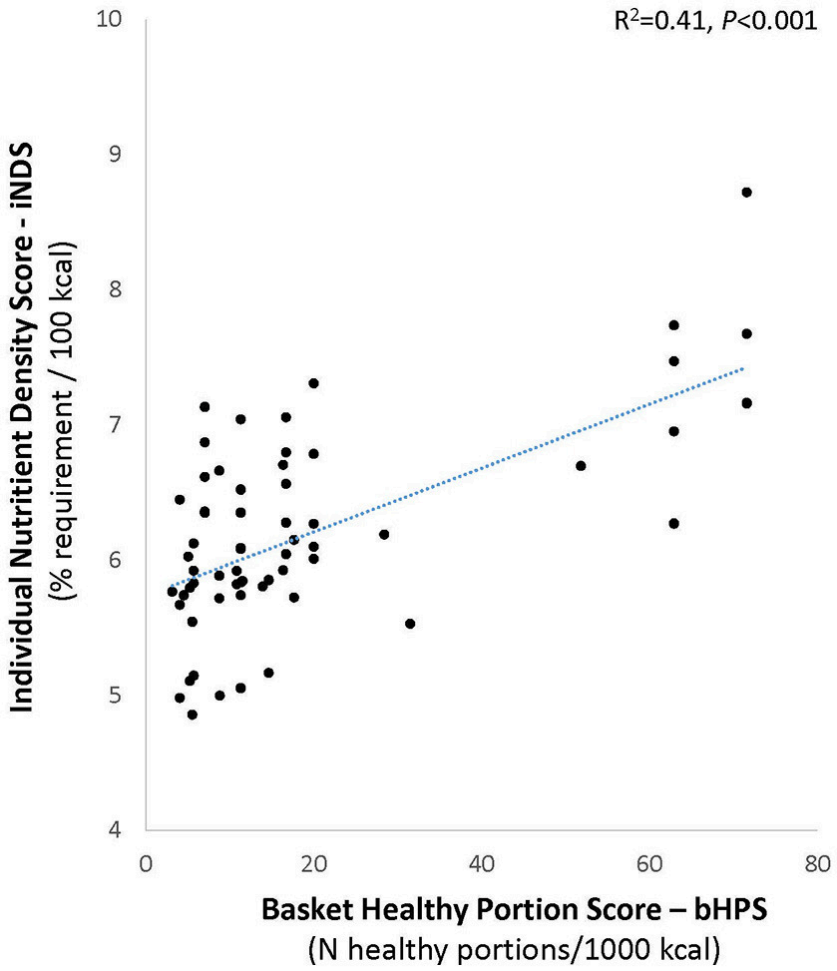
Relation between food aid quality and the quality of individual aid recipient diet. Chart shows unstandardized predicted individual nutrient density scores (iNDS) predicted by basket healthy portion scores (bHPS) in regression adjusted for gender, marital status, and country of birth.

Although the baskets provided a relatively small portion of the dietary requirements of the recipients (as shown by Table 5) the quality of food aid as indicated by the basket healthy portion score (bHPS) was positively correlated with the quality of the aid-recipients' diet (iNDS). In other words, better quality baskets were correlated with healthier diets. The unadjusted bHPS was positively correlated with the individual dietary nutrient density (iNDS, bivariate correlation coefficient R = 0.27, *p* < 0.01). In a linear regression, the correlation remained highly significant even after adjusting for gender, marital status, and country of birth (Standardized β = 0.22, *p* = 0.03. Overall model R^2^ = 0.18, *F* = 4.65, *p* < 0.01). Other significant predictors of iNDS were country of birth (Standardized β = 0.26, *p* = 0.01) and gender (Standardized β = 0.213, *p* = 0.04). Fruits and vegetable portions contributed by *Leket Israel* correlated positively with dietary quality (iNDS) after adjustment for the same covariates (Standardized β = 0.20, *p* = 0.04). Figure 5 (R^2^ = 0.41, *F* = 60.0, *p* < 0.001) clearly shows the linear relationship between bHPS and the unstandardized iNDS values predicted by this linear regression model.

As expected, the participants' subjective sense of general health was significantly correlated with most of the self-reported medical conditions (Osteoporosis, high cholesterol, triglycerides, and high blood-pressure) and very highly correlated with total number of self-reported medical conditions (*F* = 14.8, *p* ≤ 0.001). Although general health was not significantly associated with the severity of food insecurity (*F* = 2.26, *p* = 0.09), the severity of food insecurity was significantly associated the number of non-communicable diseases (NCDs, Pearson correlation coefficient = 0.25, *p* = 0.02). A self-reported history of anemia was associated with a trend for lower dietary iron intake as assessed by FFQ: The χ^2^ test shows that individuals reporting anemia were significantly more likely to have dietary iron intake below the RDA than those without a history of anemia (χ^2^ = 5.47, *p* = 0.012). Individuals with a history of anemia also received aid baskets with significantly lower Nutrient Density Scores (*t* = −2.20, *p* = 0.03). Unexpectedly, individuals who reported a diagnosis of diabetes had better diets compared to those who did not report diabetes as indicated by a higher healthy portions density (*t* = 2.30, *p* = 0.02) and higher nutrient density score (*t* = 2.69, *p* < 0.01). Qualitative data from our interviews indicated that this is probably due to lifestyle changes made by diabetics due to their condition and treatment by doctors and dieticians in the community. Other than this, basket quality was not cross-sectionally related to chronic health conditions.

## Discussion

The need for research relating the quality of food provided by food banks and pantries in affluent countries to the quality of the aid recipient's diet and health has been highlighted by recent systematic reviews and meta-analyses (20, 21). Analyzing this linkage is essential to evaluate the impact of food bank interventions. Our study addressed this important question by simultaneously assessing food-aid and individual dietary quality in a reasonably large convenience sample representing a wide variety of food pantries and social diversity.

We found a significant positive correlation between the quality of the food-aid supplied by NPOs in the Leket Israel collaborative and the quality of the aid-recipients' diet. The correlation indicates that the NPOs adherence to government guidelines regarding a healthy diet on a tight budget is associated with recipients benefiting from more nutrient dense diets, regardless of relevant demographic factors. This finding is important because it suggests that nutritionally high-quality food distribution can contribute to the diet quality of food insecure populations. This is relevant to resource allocation and policy making within NPOs, food banks, and the government.

Absent an index reflecting Israeli guidelines and realities, we devised a new score, the HPS, to assess how well food baskets and individual diets conform to Israeli government guidelines based on the “Mediterranean diet” to promote healthful, adequate nutrition even for households on a tight budget (28). This score is conceptually similar to the Healthy Eating Index (HEI) developed in the USA to assess conformance of individual diets with official government recommendations (31, 32). Our findings lend confidence to the validity and utility of using the basket HPS to inform Israeli food security policy. Its use here provides the first quantitative evidence that efforts to distribute fruit and vegetables through the supply chain from food bank through pantry to recipient yields measurable benefit for the individuals who receive the food.

An interesting observation, consistent with similar accounts in the literature (21, 33–35), is that the correlation between food-aid quality and recipient diet quality was positive despite the limited nutritional adequacy of the food baskets in the present study. The fact that, despite being inadequate, the number of healthy portions of fruits and vegetables in the food-aid correlated with a better habitual recipient diet suggests that food-aid composition may have behavioral or educational benefits beyond their inherent nutritional and economic value. The literature shows that food, vouchers, and cash transfers are not simply fungible; although they increase the recipients' purchasing power to the same extent, they can influence behavior differently, depending on factors such as the degree of poverty and accessibility and availability of nutritious food (36–39). The more the responsibility of food banks for hunger relief in affluent countries increases, the more information we need on how food aid composition influences recipient dietary behavior. This in turn will enable food banks and policy makers to optimize the quantity and quality of food-aid that will better promote recipient food security, nutrition, and health.

Such optimization is well warranted. As expected, the dietary assessment of the study population revealed significant deficits with high percentages of participants not reaching requirements for protein, calcium, vitamin D, vitamin E, folate, fiber, or government recommendations of healthy food portions. This is consistent with the literature on food pantry users elsewhere (20, 33, 40–45). As also might be expected from the international literature (15, 16, 20, 46–51) our study documented poor health among Israeli food pantry users. The prevalence of non-communicable diseases, obesity and subjective poor health were considerably higher than among the general Israeli population. For example, 84% of all Israeli adults report “good” or “very good” health in national surveys, as do 59% of older adults above age 75 (52). In contrast, only 57% of our population reported “good” or “very good” health, despite their much younger mean age of ~51 years. Data from a National Insurance Institute and Ministry of Health survey of food insecure households show that one third of surveyed households had at least one member with diet-related conditions including anemia (33%), high blood pressure (22%), diabetes (17%) and cardiovascular disease (12%). These rates are 2–3 times higher than would be expected in the general population (53). Self-reported rates of these conditions among our sample of food pantry users were even higher (Table 3). Furthermore, 34 % of our study population were classified as obese which is nearly twice the 18% prevalence in the general Israeli population (52). These findings underscore the importance of ensuring access to healthful food for this population which experiences a high burden and premature onset of diet-related diseases.

Our study has several limitations. Nearly half our survey population reported food insecurity with some degree of hunger; however, the fact that 17% of pantry users' self-report indicated that they were not food insecure points to the challenge of the subjective household food security questionnaire. The personal and invasive nature of the questions can bias the responses by evoking embarrassment or a desire to please the interviewer or through perceived harm or benefit that the participant might associate with a given response to some questions. While the interviewers' impression was that most of the respondents openly answered the questions, the incomplete response rate was rather high (Figure 1). The fact that the majority of participants reported high or very high interest in nutrition also raises the possibility of self-selection bias. Convenience recruitment by food pantries' staff might similarly entail bias, selecting for compliant participants or for those with an especially dire situation who would be more highly motivated for compensation. The purpose of this exploratory survey limited to a convenience sample of 100 individuals was to develop methodology and ascertain options for more robust follow-up research. Nevertheless, our sample size and its diversity are well within the norms for the handful of comparable studies in the literature, which also tend to be relatively small and local (20, 21). Although our sample is not strictly representative or proportional, it nevertheless captures considerable regional and ethnic diversity from the target population across the country, as well as a spectrum of contributing factors for food insecurity (i.e., low income, large household size, single breadwinner, employment status, disease and disability, age, and immigrant status). It should be noted that the FFQ was not formally validated for use in this particular—i.e., food insecure—population. It was designed for the general Israeli population but may fail to capture foods that may be uniquely consumed by Israeli Arabs, Ethiopian immigrants or other ethnic groups (26, 54). Due to the small sample size we were unable to analyze ethnic subgroups separately, but it will be important to address this question in future studies (55). While the FFQ is the best available tool for measuring habitual intake, its limitations include overestimation compared to 24-h recall questionnaires (27, 56, 57) and is considered a cognitively difficult task (58).

Future studies will need to consider additional issues that were beyond the scope of the present undertaking. Although we incorporated household size into our basket quality measures, we relied on a single adult respondent to represent household diets. Future work should address differences in food allocation and utilization between children and adult household members (19). Indeed, some participants in our study remarked that they gave any food-aid to their children, placing their children's needs before their own; however, we could not address this issue quantitatively in the present study. It will also be interesting to probe household economics more deeply: How much of the limited income is allocated to different needs and what are their trade-offs with the procurement of food? What are the other sources of food in the household? How do families reorganize to deal with food insecurity? How does nutritional knowledge affect food purchase choices? Consideration of food prices should be incorporated in future analyses for the development of indices which score food baskets and diets not only by their nutritional quality, but also by their cost and accessibility. Future studies may use our survey approach to examine the cultural appropriateness of the food aid in light of the experience and attitudes of the recipients.

Empirical research alone cannot answer the larger question of whether food banks are an appropriate or ethical response to food insecurity and poverty in affluent societies. Nevertheless, data are vital to inform and guide the policy debate as charitable food banks become increasingly embedded in national responses to poverty and hunger. While cross-sectional research like the present study can demonstrate association but not causation, methods and measures similar to those we developed here can be used in future prospective observational studies and perhaps even randomized interventions to assess the potential drawbacks and benefits of efforts to mitigate food insecurity and accompanying ill health. Data on how food banks influence their users' health is crucial to inform practice and policies that not only alleviate immediate hunger, but contribute effectively and ethically to the long-term health and wellbeing essential to escaping the downward spiral of poverty. This study represents a necessary step in that direction.

## Author Contributions

DE and AT designed the research. DE and GB conducted the survey. DE and DS analyzed the data. DE, DS, and AT wrote the manuscript and AT has overall responsibility for the study.

### Conflict of Interest Statement

Funding for the research was provided by a grant from Leket Israel. Leket Israel had no part in the design, analysis, interpretation and write-up of this study. The authors declare that the research was carried out in the absence of any other percieved or actual commercial or financial conflicts of interest. The findings and their interpretation are the authors alone. The handling Editor declared a shared affiliation, though no other collaboration, with several of the authors DE, GB, and AT.

## References

[1] Ashton JR Middleton J Lang T. Open letter to Prime Minister David Cameron on food poverty in the UK. Lancet (2014) 383:1631. 10.1016/S0140-6736(14)60536-5 24794817

[2] Loopstra R Reeves A Taylor-Robinson D Barr B McKee M Stuckler D. Austerity, sanctions, and the rise of food banks in the UK. BMJ (2015) 350:h1775. 10.1136/bmj.h1775 25854525

[3] Poppendieck J Sweet Charity?: Emergency Food and the End of Entitlement. New York, N.Y: Viking (1998).

[4] Tarasuk V Eakin JM Food assistance through “surplus” food: Insights from an ethnographic study of food bank work. Agri Hum Values (2005) 22:177–86. 10.1007/s10460-004-8277-x

[5] Winne M Closing the Food Gap: Resetting the Table in the Land of Plenty. Boston, MA: Beacon Press (2008).

[6] Caraher M Coveney J editors. Food Poverty and Insecurity: International Food Inequalities. Cham: Springer (2016).

[7] Riches G Food Banks and Food Security: Welfare Reform, Human Rights and Social Policy. Lessons from Canada? Soc Policy Admin. (2002) 36:648–63. 10.1111/1467-9515.00309

[8] Campbell EC Ross M Webb KL Improving the nutritional quality of emergency food: a study of food bank organizational culture, capacity, and practices. J Hunger Environ Nutr. (2013) 8:261–80. 10.1080/19320248.2013.816991

[9] Philip D Hod-Ovadia S Troen AM. A technical and policy case study of large-scale rescue and redistribution of perishable foods by the “Leket Israel” food bank. Food Nutr Bull. (2017) 38:226–39. 10.1177/0379572117692440 28513266

[10] Bhattacharyaa JCJ Haider S Poverty, food insecurity, and nutritional outcomes in children and adults. J Health Econ. (2004) 23:839–62. 10.1016/j.jhealeco.2003.12.008 15587700

[11] Chernichovsky D Regev E Patterns of expenditure on food in Israel. Taub Center for Social Policy Studies in Israel. (2014) Available online at: http://taubcenter.org.il/wp-content/files_mf/e2014.16patternsoffoodexpenditure79.pdf

[12] Kirkpatrick SI Tarasuk V. Food insecurity is associated with nutrient inadequacies among Canadian adults and adolescents. J Nutr. (2008) 138:604–12. 10.1093/jn/138.3.604 18287374

[13] Cook JT BM Chilton M Cutts D Ettinger de Cuba S Heeren TC Rose-Jacobs R. Are food insecurity's health impacts underestimated in the U.S. population? marginal food security also predicts adverse health outcomes in young, U.S. children and mothers. Adv Nutr (2013) 4:51–61. 10.3945/an.112.003228 23319123 PMC3648739

[14] Lyles CR WM Schillinger D Davis TC DeWalt D Dahlke AR Curtis L Seligman HK. Food insecurity in relation to changes in hemoglobin A1c, self-efficacy, and fruit/vegetable intake during a diabetes educational intervention. Diabetes Care (2013) 36:1448–53. 10.2337/dc12-1961 23275354 PMC3661820

[15] Park CY Eicher-Miller HA. Iron deficiency is associated with food insecurity in pregnant females in the United States: National Health and Nutrition Examination Survey 1999–2010. J Acad Nutr Diet. (2014) 114:1967–73. 10.1016/j.jand.2014.04.025 24953790

[16] Seligman HK Kushel MB. Food insecurity is associated with chronic disease among low-income NHANES participants. J Nutr. (2010) 140:304–10. 10.3945/jn.109.112573 20032485 PMC2806885

[17] Tarasuk V Cheng J de Oliveira C Dachner N Gundersen C Kurdyak P. Association between household food insecurity and annual health care costs. CMAJ (2015) 187:E429–E36. 10.1503/cmaj.150234 26261199 PMC4592315

[18] Barrett CB Measuring food security. Science (2010) 327:825–8. 10.1126/science.1182768 20150491

[19] Webb P Coates J Frongillo EA Rogers BL Swindale A Bilinsky P Measuring household food insecurity: why it's so important and yet so difficult to Do1. J Nutr. (2006) 136:1404S−8S. 10.1093/jn/136.5.1404S 16614437

[20] Simmet A Depa J Tinnemann P Stroebele-Benschop N. The dietary quality of food pantry users: a systematic review of existing literature. J Acad Nutr Diet. (2017) 117:563–76. 10.1016/j.jand.2016.08.014 27727100

[21] Simmet A Depa J Tinnemann P Stroebele-Benschop N. The nutritional quality of food provided from food pantries: a systematic review of existing literature. J Acad Nutr Diet. (2017) 117:577–88. 10.1016/j.jand.2016.08.015 27727101

[22] Endeweld M Barkali N Abrahamov V Gealia A Gottlieb D 2012 food security survey main socio-economic findings. In: Research Report #115. National Insurance Institute, Research and Planning Administration. Jerusalem (2014). Available online at: https://www.btl.gov.il/Publications/research/Pages/mechkar_115.aspx

[23] Chernichovsky D Zuk-Bar U Harmati G Cohen Z Madar Z Hovav H National Program to Ensure Household Food Security in Israel (Hebrew). In. National Nutritional Security Council Report, Jerusalem (2014). Available online at: http://www.ozdov.com/articles-publications/item/download/111_034ceeaa6be4f5bb615d40fa99749154

[24] Bickel GG Nord M Price C Hamilton W Cook J Evaluation Guide to Measuring Household Food Security, Revised 2000. U.S. Department of Agriculture, Food Nutrition Service, Alexandria, VA (2000). Available online at: https://www.fns.usda.gov/guide-measuring-household-food-security-revised-2000

[25] Israel Center for Disease Control MABAT: First Israeli National Health Nutrition Survey 1999–2001. Ministry of Health. Jerusalem. (2003). Available online at: https://www.health.gov.il/PublicationsFiles/Mabat_1999-2001-a.pdf

[26] Shahar D Vardi H Brener-Azrad A Fraser D. Development of a semi-quantitative Food Frequency Questionnaire (FFQ) to assess dietary intake of multiethnic populations. Eur J Epidemiol. (2003) 18:855–61. 10.1023/A:1025634020718 14561044

[27] Willett W. Food frequency methods. In: Willet W. editor. Nutritional Epidemiology. Oxford Scholarship Online. Oxford: Oxford University Press (2013).

[28] Azarieva J Orion B Goldsmith R Chernichovsky D A Healthy Food Basket in Israel (Hebrew). Jerusalem: Taub Center for Social Policy Studies in Israel (2016).

[29] Darmon N Darmon M Maillot M Drewnowski A. A nutrient density standard for vegetables and fruits: nutrients per calorie and nutrients per unit cost. J Am Diet Assoc. (2005) 105:1881–7. 10.1016/j.jada.2005.09.005 16321593

[30] Drewnowski A. Defining nutrient density: development and validation of the nutrient rich foods index. J Am Coll Nutr. (2009) 28:421S−6S. 10.1080/07315724.2009.10718106 20368382

[31] Guenther PM Casavale KO Reedy J Kirkpatrick SI Hiza HA Kuczynski KJ. Update of the healthy eating index: HEI-2010. J Acad Nutr Diet. (2013) 113:569–80. 10.1016/j.jand.2012.12.016 23415502 PMC3810369

[32] Guenther PM Reedy J Krebs-Smith SM Reeve BB Basiotis PP Development Evaluation of the Healthy Eating Index-2005. Technical Report. Center for Nutrition Policy Promotion, U.S. Department of Agriculture (2007). Available online at: http://www.cnpp.usda.gov/HealthyEatingIndex.htm.

[33] Irwin JD Ng VK Rush TJ Nguyen C He M. Can food banks sustain nutrient requirements? A case study in Southwestern Ontario. Can J Public Health (2007) 98:17–20. 10.17269/cjph.98.802 17278671 PMC6975872

[34] Neter JE Dijkstra SC Visser M Brouwer IA Dutch food bank parcels do not meet nutritional guidelines for a healthy diet. Br J Nutr (2016) 116:526–33. 10.1017/S0007114516002087 27229880

[35] Akobundu UO Cohen NL Laus MJ Schulte MJ Soussloff MN. Vitamins A and C calcium, fruit, and dairy products are limited in food pantries. J Am Diet Assoc (2004) 104:811–3. 10.1016/j.jada.2004.03.009 15127070

[36] Basu K Relief programs: when it may be better to give food instead of cash. World Dev. (1996) 24:91–6. 10.1016/0305-750X(95)00110-X

[37] Gentilini U Cash Food Transfers: A Primer. World Food Program Rome (2007). Available online at: http://www.wfp.org/content/cash-and-food-transfers-primer-ugo-gentilini-2007

[38] Meyerhoefer CD Yang M The relationship between food assistance and health: a review of the literature and empirical strategies for identifying program effects. Appl Econ Perspect Policy (2011) 33:304–44. 10.1093/aepp/ppr023

[39] Michelson H Lentz EC Mulwa R Morey M Cramer L McGlinchy M Cash, food or vouchers in urban and rural Kenya? An application of the market information and food insecurity response analysis framework. Food Secur. (2012) 4:455–69. 10.1007/s12571-012-0177-0

[40] Jacobs Starkey L Gray-Donald K Kuhnlein HV. Nutrient intake of food bank users is related to frequency of food bank use, household size, smoking, education and country of birth. J Nutr. (1999) 129:883–9. 10.1093/jn/129.4.883 10203565

[41] Jacobs Starkey L Kuhnlein HV. Montreal food bank users' intakes compared with recommendations of Canada's food guide to healthy eating. Can J Diet Pract Res. (2000) 61:73–5. 11551351

[42] Neter JE Dijkstra SC Dekkers ALM Ocke MC Visser M Brouwer IA. Dutch food bank recipients have poorer dietary intakes than the general and low-socioeconomic status Dutch adult population (2017). Eur J Nutr. 57:2747–58. 10.1007/s00394-017-1540-x 28975454 PMC6267415

[43] Rush TJ Ng V Irwin JD Stitt LW He M. Food insecurity and dietary intake of immigrant food bank users. Can J Diet Pract Res. (2007) 68:73–8. 10.3148/68.2.2007.73 17553192

[44] Teron AC Tarasuk VS. Charitable food assistance: what are food bank users receiving? Can J Public Health (1999) 90:382–4. 10680260 10.1007/BF03404139 PMC6980020

[45] Duffy P Zizza C Jacoby J Tayie FA. Diet quality is low among female food pantry clients in eastern Alabama. J Nutr Educ Behav. (2009) 41:6. 10.1016/j.jneb.2008.09.002 19879497

[46] Leung CW Epel ES Willett WC Rimm EB Laraia BA. Household food insecurity is positively associated with depression among low-income supplemental nutrition assistance program participants and income-eligible nonparticipants. J Nutr. (2015) 145:622–7. 10.3945/jn.114.199414 25733480

[47] Long CR Rowland B Steelman SC McElfish PA. Outcomes of disease prevention and management interventions in food pantries and food banks: protocol for a scoping review. BMJ Open (2017) 7:e018022. 10.1136/bmjopen-2017-018022 28982837 PMC5640003

[48] Melchior M Chastang J-F Falissard B Galera C Tremblay RE Cote SM. Food insecurity and children's mental health: a prospective birth cohort study. PLoS ONE 7:e52615. 10.1371/journal.pone.0052615 23300723 PMC3530436

[49] Olson CM. Nutrition and health outcomes associated with food insecurity and hunger. J Nutr. (1999) 129:521S−4S. 10.1093/jn/129.2.521S 10064322

[50] Weiser SD Palar K Hatcher AM Young S Frongillo EA Laraia B Food insecurity and health a conceptual framework. In: Ivers L, editor. Food Insecurity and Public Health. 1 ed. CRC Press (2015). p. 23–50.

[51] Institute of Medicine (2011) Hunger and Obesity: Understanding a Food Insecurity Paradigm: Workshop Summary. Washington, DC: The National Academies Press. 24983070

[52] Israel Center for Disease Control Israel National Health Interview Survey INHIS-3, 2013–2015 – Selected Findings. Ministry of Health, Jerusalem (2017). Available online at: https://www.health.gov.il/PublicationsFiles/INHIS_3.pdf

[53] Endeweld M Goldsmith R Endevelt R. The demographic and morbidity characteristics of a population receiving food support in Israel. Israel J Healtj Policy Res. (2018) 7:54. 10.1186/s13584-018-0238-8 30165905 PMC6389190

[54] Shai I Shahar D Vardi H Fraser D. Selection of food items for inclusion in a newly developed food-frequency questionnaire. Public Health Nutr. (2004) 7:745–9. 10.1079/PHN2004599 15369612

[55] Abu-Saad K Shahar DR Vardi H Fraser D. Importance of ethnic foods as predictors of and contributors to nutrient intake levels in a minority population. Eur J Clin Nutr. (2010) 64(Suppl 3):S88–94. 10.1038/ejcn.2010.217 21045858

[56] Bogers RP Dagnelie PC Westerterp KR Kester AD van Klaveren JD Bast A Using a correction factor to correct for overreporting in a food-frequency questionnaire does not improve biomarker-assessed validity of estimates for fruit and vegetable consumption. J Nutr. (2003) 133:1213–9. 10.1093/jn/133.4.1213 12672945

[57] Smiciklas-Wright H Mitchell DC Wheeler D Dietary intake assessment methods for adults. In: Berdanier CD Dwyer JT Heber D, editors. Handbook of Nutrition and Food, Third Edition. Boca Raton: CRC Press (2014). p. 517–30.

[58] Shim J-S Oh K Kim HC. Dietary assessment methods in epidemiologic studies. Epidemiol Health (2014) 36:e2014009. 10.4178/epih/e2014009 25078382 PMC4154347

