# Supplementary material for: Food-Aid Quality Correlates Positively With Diet Quality of Food Pantry Users in the Leket Israel Food Bank Collaborative
Source: Front Nutr. 2018 Dec 18;5:123. doi: 10.3389/fnut.2018.00123 (PMC6305620; doi:10.3389/fnut.2018.00123)
Supplement: Supplementary file 1 [file Presentation_1.pdf]

Online Supporting Information

Food-Aid Quality is Positively Correlated With Diet Quality Among Food Pantry Users In The *Leket Israel* Food Bank Collaborative

Dana Efrati Philip[1], Ghada Baransi[1], Danit R. Shahar[2], Aron M. Troen, D. Phil[1]

[1]The Nutrition and Brain Health Laboratory, The Institute of Biochemistry Food Science and Nutrition, The Robert H. Smith Faculty of Agriculture, Food and Environment, The Hebrew University of Jerusalem

[2]The S. Daniel Abraham International Center for Health and Nutrition, Department of Public Health, Faculty of Health Sciences, Ben-Gurion University of the Negev

**Expanded Methods: NNSC Healthy Basic Food Basket Tables**	**p. 2**
**Expanded Methods: Detailed derivation of nutritional quality scores**	**p. 3-5**
**Structured Survey Questionnaire (Hebrew)**	**p. 6-10**
**Food Frequency Qustionnaire (Hebrew)**	**p. 11-16**
**Structured Survey Questionnaire (Arabic)**	**p. 17-23**
**Food Frequency Qustionnaire (Arabic)**	**p. 24-30**

NNSC Healthy Basic Food Basket Tables

Table 1. Details of the foods in the various food groups

Food group	Details of foods included in the group	Foods not included in the group
Whole grains	Whole wheat bread, pita, noodles, potatoes, rice	Breakfast cereals, baked goods, crackers
Fruit and vegetables	Fresh and cooked vegetables and fruit	Fresh juice, dried fruit
Protein-rich foods	Milk, yogurt, white and yellow cheese, meat, chicken, turkey, fish, eggs, legumes	Puddings, ice cream, cream, peanuts, sunflower and other seeds, fatty meats
Foods high in fat	Oil, nuts, avocado, tehina	Margarine, butter and foods high in fat and transfats

Source: Ministry of Health, Department of Nutrition.

Table 2. Daily recommended nutritional servings

By gender and age

Age (years) and gender	Recommended caloric intake	groups					
		Whole grains	Vegetables	Fruit	Protein-rich foods		Fatty foods
					Milk	Animal/ legumes	
2-3*	1,300	6	3	2	1	3	1
4-6	1,800	7	3	2	1	3	1
7-10	2,000	8	4	3	1	3	1.5
11-14	2,500	10	5	4	2	4	3
15-18 (boys)	3,000	11	5	4	2	4	3
19-24 (boys)	2,900	11	5	4	1	4	3
25-50 (men)	2,900	11	5	4	2	4	3
51 + (men)	2,300	9	4	3	2	3	3
11-24 (women)	2,200	9	4	3	2	3	3
25-50 (women)	2,200	9	4	3	2	3	3
51+ (women)	1,900	7	4	3	2	2	3

* Serving size for ages 2-3 are equal to 2/3 of the serving size for the other age groups, except for milk products.

Source: Minsitry of Health, Department of Nutrition.

Detailed derivation of nutritional scores

Individual Healthy Portions Score (iHPS)

We derived the number of healthy food portions provided by the participants' habitual diets as reported on the FFQ, as well as for the contents of each food basket. Healthy portions were defined according to the "Basic Healthy Food Basket Guidelines" recommended by the Government of Israel's National Nutritional Security Council (NNSC) in cooperation with the Israeli Ministry of Health. These guidelines include 5 main food groups: whole grains, fruits, vegetables, protein-rich foods, and fats and oils. The protein-rich group is further divided into meat, dairy, and legumes. The guidelines define adequate portion sizes for each of these groups and the number of recommended daily portions for individual consumption according to age and sex (see online supporting information for further detail).

To derive the individual healthy portion score (iHPS) we first assigned any healthy FFQ items to their corresponding healthy food group as defined by the guidelines, with the following additional conditions:

1. Whole grains and potatoes were entered to the whole grain group only when explicitly consumed as whole food. French fries were excluded.
2. Only low processed, unsweetened vegetables were included in the vegetables group. Thus, for example, sweet canned corn was excluded from the healthy portions.
3. Industrial manufactured hummus was not included a legume.

Second, we calculated the number of portions contributed by each FFQ item as follows (**Formula a** below):

1. If the FFQ item portion-sizes were based on weight – as was the case with the whole grain, vegetable, fruit, and protein-rich food groups – we converted the portion-size of each group into grams (using the USDA food database(23); $portion_{wt}$ in formula a.) and divided the edible food weight of each item ($weight_i$ in formula a) by its equivalent portion-size.
2. Where portion sizes were based on the amount of a nutrient – dairy portions were based on calcium(mg) and fat portions – on fat(g) – we calculated the number of portions by dividing the amount of the relevant nutrient in that food item ($calcium_i$ or fat_i) by the recommended nutrient-portion size ($portion_{Ca}$ or $portion_{fat}$).

Formula a. The number of portions for a given FFQ food item:

$$\frac{weight_{i}}{portion_{wt}}\ OR\ \frac{calcium_{i}}{portion_{Ca}}\ OR\ \frac{fat_{i}}{portion_{fat}}$$

Third, for each food group, we summed the total number of healthy portions contributed by each included FFQ item and aggregated the number of food portions by participant. Finally, we divided the total number of healthy portions by total energy intake ($energy_{total}$, kcal) as recorded in the FFQ (**Formula b)**. This was done in order to account also for the possible negative effect of unhealthy foods in the diet. This measure integrates quantity and quality with a magnifying effect by healthy portions and a lessening effect by overall energy content.

Formula b. Individual Healthy Portions Score:

$$\frac{portion_{\text{grains}} + portion_{\text{fruit}} + portion_{\text{veg}} + portion_{\text{meat}} + portion_{\text{dairy}} + portion_{\text{fat}}}{energy_{\text{total}}}$$

Individual Nutrient Density Score (iNDS)

The Individual Nutrient Density Score was calculated as previously described by Drewnowski (24,25). First, we calculated the Individual Nutrient Adequacy Score (iNAS) for each FFQ by dividing the content of 16 nutrients in the diet by the individual RDA requirements (iDV_i) by age and sex (**Formula c**). The average of these 16 ratios between nutrient amount in diet and RDA was divided by 100gr edible weight. Second, the individual Energy Density Score (iEDS, **Formula d**) was calculated by dividing the energy content (kcals) by 100gr edible weight. Finally, the iNAS was divided by the iEDS to yield the individual Nutrient Density Score (iNDS, **Formula e**). This score can be used as an index of the adequacy and density of both foods and diets according to their content of 16 main nutrients and energy.

Formula c. Individual Nutrient Adequacy Score (iNAS):

$$\frac{\sum \{ Nutrient_{\text{i}} \ / \ iDV_{\text{i}} \} \ X \ 100}{16} X \frac{100}{iWeight}$$

Formula d. Individual Energy Density (iEDS):

$$\frac{iEnergy}{iWeight} X \ 100$$

Formula e. Individual Nutrient Density Score (iNDS):

$$\frac{iNAS}{iEDS} X \ 100$$

Food-aid (basket) quality assessment

The contents of typical food baskets were recorded using a structured questionnaire that was provided to NPO staff. Where items were not distributed on a weekly basis, the amounts were normalized to equal weekly distribution. The total nutrient content of the food basket was extracted using the Ministry of Health's TZAMERET nutrient database and BINAT software for entering nutrition data.

Basket Healthy Portions Score (bHPS)

The number of healthy portions provided by each food basket was calculated in a similar manner to the individual score, namely: Each of the items in the food basket was assigned as appropriate to its corresponding food group according to the NNSC's "Basic Healthy Food Basket Guidelines" as described for individual diets above. Items with corresponding version in the FFQ were allocated to the same groups and if there was not a similar item in the FFQ, they were left out of the calculations in order to maintain consistency.

We calculated the number of portions contributed by each item, either by dividing edible weight by the recommended portion size (see **Formula a**) or by dividing the amount of the relevant nutrient recommended portion by its content in that food item. Then we summed up the contributed portions by the different food items for each food group and aggregated by food basket. Finally, all the contributed

portions from the 5 food groups were summed and the sum was divided by the total energy contributed by all food items in the basket. It is important to note that the bHPS score is for the weekly amount of food that is distributed to an entire household.

Basket Nutrient Density Score

For the basket nutrient density score, the total content of 16 nutrients was calculated using the TZAMERET software, as described above. For each participant, we calculated a household Recommended Weekly Allowance (hRWA) which took into consideration the dietary requirements for all members of the participants household, according to the information provided by the participant regarding their ages and gender. For each nutrient, we summed the RDAs required for the entire household and multiplied by 7 (since the food basket contents are normalized by weekly distribution). We then calculated the Basket Nutrient Adequacy Score (bNAS) for each FFQ by dividing the content of 16 nutrients in the basket by the hRWA (see **Formula f**). The average of these 16 ratios between nutrient amount in the basket and hRWA was divided by 100gr edible weight. We next calculated the basket Energy Density Score (bEDS, **Formula g**) by dividing the energy content (kcals) by 100gr edible weight. Finally, the bNAS was divided by the bEDS to yield the basket Nutrient Density Score (bNDS, **Formula h**). This score reflects the adequacy and density of the foods and diets according to the content of 16 main nutrients and energy.

Formula f. Basket Nutrient Adequacy Score:

$$\frac{\sum \{ Nutrient_{i} \,/\, hRWA_{i} \} \, X \, 100}{16} X \frac{100}{bWeight}$$

Where household recommended weekly allowance (hRWA) takes into account the RDAs for each household member according to their age and gender, and where "weight" is edible weight of the food.

Formula g. Basket Energy Density:

$$\frac{bEnergy}{bWeight} X \, 100$$

Formula h. Basket Nutrient Density Score:

$$\frac{bNAS}{bEDS} X \, 100$$

Structured Telephone Survey

שם הסוקר: __________ intname

הראיון התקיים בתאריך:_______ date שעת התחלה: ______ timestart

מספר נדגם _______ subjectID עמותה ________ npo

במידה ופוצל: תאריך המשך: _________ שעת התחלה: שעת סיום:

תאריך המשך: __________ שעת התחלה: שעת סיום:

לסוקר: קרא – "שלום, שמי ________ ואני מתקשר בנוגע להשתתפות במחקר בנושא ביטחון תזונתי. נתת את הסכמתך שיצרו אתך קשר היום בשעה זו, האם נוכל לקיים/להמשיך את הראיון?"

כן – ממשיכים. לא – האם נוכל לקיים במועד אחר? כן, איזה מועד ? יש לרשום יחד עם פרטי איש הקשר.

במידה ופוצל: מתי ביקש להמשיך? _________________ ____________________ __________________

לסוקר קרא – "אבקש להזכירך כי שאלון זה הנו אנונימי ומהווה חלק ממחקר. אי הסכמה להשתתף תתבטא באי מילוי השאלון. הנך רשאי להשיב על חלק מהשאלות בלבד. את/ה חופשי/ה להפסיק בכל עת את השתתפותך בניסוי, בלי לפגוע בזכויותיך, בלי שיאונה לך כל רע, ובלי שתינקט נגדך סנקציה כלשהי, כולל הזכות לקבלת סיוע מהעמותה. אם את/ה מרגיש/ה חשש או אי-נוחות, אנא אמר/י לי."

1. האם את/ה מסכימ/ה להמשיך בראיון? ❑ כן=1 ❑ לא=0 consent

לסוקר- כאשר אתם מוכנים להתחיל בראיון, קרא- "היות וישנם אנשים רבים שלא התנסו בעבר בראיון מסוג זה, ברשותך, תן לי להסביר בקצרה את צורת הראיון. אני עומדת לקרוא סדרת שאלות בדיוק לפי ניסוחן. אתה תתבקש לענות בשתי צורות: בחלק מהשאלות, תוכל להשיב במילים שלך ובחלק תצטרך לבחור תשובה אחת המתאימה ביותר לך, מתוך מספר אפשרויות שאקרא בפניך. ישנן שאלות שבהן ניתן לבחור יותר מתשובה אחת מתאימה. בכל שלב במהלך השאלון, אם אתה מרגיש שהשאלה לא ברורה לך, אנא תאמר לי זאת."

2. מהי שנת הלידה שלך/ך? _______ birthyear

3. מין: ❑ נקבה=1 ❑זכר=2 sex

פרטים דמוגרפיים על הנדגם

לסוקר קרא- "כעת אשאל אותך כמה שאלות כלליות על עצמך ועל משפחתך".

4. בן כמה אתה? _______ (סוקר: שים לב שזה מתאים לשנת לידה) age

5. באיזו ארץ נולדת? _________ישראל________ country

6. **באיזו שנה עלית לארץ**? ________ immigyear

7. **מה מצבך המשפחתי?**

❑ רווק/ה=1 ❑ נשוי/ה=2 ❑ גרוש/ה=3 ❑ אלמן/ה=4 ❑ חי/ה בנפרד=5

marstat אחר, פרט _______________. marstat_other

8. האם אתה:

❑ יהודי=1 ❑ ערבי מוסלמי=2 ❑ ערבי נוצרי=3 ❑ דרוזי=4 ❑ נוצרי=5
ethnicity אחר, פרט _______________. ethnicity_other

9. האם בשלושת החודשים האחרונים את/ה:

❑ לא עובד בגלל מחלה/נכות/מוגבלות=1 ❑ עובד כשכיר=2 ❑ בחופשת לידה=3
❑ עובד כעצמאי=4 ❑ גימלאי=5 ❑ סטודנט שעובד=6 ❑ לא
עובד/מובטל=7 ❑ עובד כבן משפחה ללא תשלום=8 ❑ עקרת בית=9 ❑ סטודנט
(ואינך עובד)=10 ❑ חבר קיבוץ=11 ❑ לא עובד מסיבה אחרת=12 employ

אחר, פרט __________________.employ_other

10. מהי ההכנסה החודשית של משק הבית נטו מהעבודה? _______ לא יודע/מסרב=n/a income

11. כמה אנשים מתגוררים בבית בדרך קבע (לא כולל דייר בשכירות, חייל בחובה, סטודנט שגר במעונות או מחוץ לבית)? ____________ hhn

.12 **פרט את מספר האנשים הנוספים המתגוררים בבית לפי קירבה אליך, מינם וגילם. לדוגמא: בן/בת זוג, הורה, סב/סבתא, בן/בת, אח/אחות, שותף/חבר, אחר (בטבלה)**

מספר (מלבד המרואיין)	קרבה	מין	גיל
2	hh2	hh2_sex	hh2_age
3	hh3	hh3_sex	hh3_ age
4	hh4	hh4_sex	hh4_ age
5	hh5	hh5_sex	hh5_ age
6	hh6	hh6_sex	hh6_ age
7	hh7	hh7_sex	hh7_ age
8	hh8	hh8_sex	hh8_ age
9	hh9	hh9_sex	hh9_ age
10	hh10	hh10_sex	hh10_ age

❑בן/בת זוג=1 ❑ הורה=2 ❑ סב/סבתא=3 ❑ בן/בת=4 ❑ אח/אחות=5
❑ שותף/חבר=6 ❑ אחר=7

לסוקר: קרא- "בשאלות הבאות אבקש ממך פרטים על צריכת המזון שלך. אקריא רשימה של מוצרי מזון ועבור כל מוצר אשאל באיזו תדירות את/ה צורך את אותו המוצר."

(עבור לשאלון FFQ)

שאלון ביטחון תזונתי – יש לשים לב האם השאלות מיועדות למשפחות אם ילדים או לא (שאלות 16-18,26-30 מיועדות למשפחות עם ילדים בלבד):

לסוקר- קרא: "אקריא לך כמה משפטים שאמרו אנשים על מצב המזון אצלם בבית. לגבי כל משפט, אמור לי בשנה האחרונה, האם לך ולבני ביתך הוא היה לרוב נכון, לפעמים נכון או בכלל לא נכון."

.13 "חששנו (חששתי) שנגמור את האוכל לפני שיהיה לנו כסף לקנות עוד."

❑ לרוב נכון=1 ❑ לפעמים נכון =2 ❑ לא נכון בכלל =3 ❑ לא יודע =4
❑ מסרב לענות =5 fs1

.14 "האוכל שקנינו לא הספיק, ולא היה לנו כסף כדי לקנות יותר."

❑ לרוב נכון=1 ❑ לפעמים נכון =2 ❑ לא נכון בכלל =3 ❑ לא יודע =4
❑ מסרב לענות =5 fs2

15. "לא היה לנו מספיק כסף לאכול ארוחות מאוזנות."

❑ לרוב נכון=1 ❑ לפעמים נכון =2 ❑ לא נכון בכלל =3 ❑ לא יודע =4
❑ מסרב לענות =5 fs3

16. "סמכנו על מספר מזונות זולים כדי להאכיל את הילדים, כי הכסף שעמד לרשותנו הלך ונגמר" (שאלה למשפחות עם ילדים)

❑ לרוב נכון=1 ❑ לפעמים נכון =2 ❑ לא נכון בכלל =3 ❑ לא יודע =4
❑ מסרב לענות =5 fs4

17. "לא היה באפשרותנו לתת לילדים ארוחות מאוזנות כי לא היה לנו כסף." (שאלה למשפחות עם ילדים)

❑ לרוב נכון=1 ❑ לפעמים נכון =2 ❑ לא נכון בכלל =3 ❑ לא יודע =4
❑ מסרב לענות =5 fs5

18. "הילדים לא אכלו מספיק כי לא יכולנו לקנות מספיק אוכל" (שאלה למשפחות עם ילדים)

❑ לרוב נכון=1 ❑ לפעמים נכון =2 ❑ לא נכון בכלל =3 ❑ לא יודע =4
❑ מסרב לענות =5 fs6

19. בשנה האחרונה, האם אתה או מבוגרים אחרים בבית צמצמו בגודל הארוחות או דילגו על ארוחות מחוסר כסף לקנות מזון?

❑ כן =1 ❑ לא = 2 ❑ לא יודע =3 ❑ מסרב לענות =4 fs7

20. באיזה תדירות זה קורה?

❑ כמעט כל חודש=1 ❑ חודשים כן, חודשים לא =2 ❑ רק חודש אחד או חודשיים במשך השנה =3
❑ לא יודע =4 ❑ מסרב לענות =5 fs8

21.האם בשנה האחרונה אכלתם פחות ממה שרציתם כי לא היה מספיק כסף כדי לקנות אוכל?

❑ כן =1 ❑ לא = 2 ❑ לא יודע =3 ❑ מסרב לענות =4 fs9

22. האם בשנה האחרונה אתה ומבוגרים אחרים בבית הייתם רעבים ולא אכלתם כי לא היה לכם מספיק כסף לקנות אוכל?

❑ כן =1 ❑ לא = 2 ❑ לא יודע =3 ❑ מסרב לענות =4 fs10

23. האם בשנה האחרונה אתה ומבוגרים אחרים בבית ירדתם במשקל כי לא היה לכם מספיק כסף לקנות אוכל?

❑ כן =1 ❑ לא = 2 ❑ לא יודע =3 ❑ מסרב לענות =4 fs11

24. במהלך השנה האחרונה, האם אתה ומבוגרים אחרים בבית לא אכלתם יום שלם כי לא היה מספיק כסף לאוכל?

❑ כן =1 ❑ לא = 2 ❑ לא יודע =3 ❑ מסרב לענות =4 fs12

25. באיזה תדירות זה קורה?

❑ כמעט כל חודש=1 ❑ חודשים כן, חודשים לא =2 ❑ רק חודש אחד או חודשיים במשך השנה =3
❑ לא יודע =4 ❑ מסרב לענות =5 fs13

26. האם בשנה האחרונה צמצמתם בגודל הארוחות של הילד/ים כי לא היה מספיק כסף לאוכל? (שאלה למשפחות עם ילדים)

❑ כן =1 ❑ לא = 2 ❑ לא יודע =3 ❑ מסרב לענות =4 fs14

27. האם בשנה האחרונה הילד/ים דילגו על ארוחות כי לא היה מספיק כסף לאוכל? (שאלה למשפחות עם ילדים)

❑ כן =1 ❑ לא = 2 ❑ לא יודע =3 ❑ מסרב לענות =4 fs15

28. באיזה תדירות זה קורה? (שאלה למשפחות עם ילדים)

❑ כמעט כל חודש=1 ❑ חודשים כן, חודשים לא =2 ❑ רק חודש אחד או חודשיים במשך השנה =3
❑ לא יודע =4 ❑ מסרב לענות =5 fs16

29. האם בשנה האחרונה הילד/ים שלך היה/היו רעב/ים ולא יכולת לקנות עוד אוכל? (שאלה למשפחות עם ילדים)

❑ כן =1 ❑ לא = 2 ❑ לא יודע =3 ❑ מסרב לענות =4 fs17

30. במהלך השנה האחרונה, האם הילדים בבית לא אכלו במשך יום שלם כי לא היה מספיק כסף לאוכל? (שאלה למשפחות עם ילדים)

❑ כן =1 ❑ לא = 2 ❑ לא יודע =3 ❑ מסרב לענות =4 fs18

סוקר: קרא- "בשאלות הבאות אשאל לגבי עמדותיך בנושאים הקשורים לתזונה."

31. באיזו מידה אתה מתעניין בקשר שבין תזונה ובריאות?

❑ במידה רבה מאד=1 ❑ במידה רבה=2 ❑ במידה מועטה=3 ❑ בכלל לא=4
❑ לא יודע=5 attitude1

32. באיזו מידה הרגלי התזונה שלך מושפעים ממידע או מפרסומים לגבי הקשר שבין תזונה ובריאות?

❑ במידה רבה מאד=1 ❑ במידה רבה=2 ❑ במידה מועטה=3 ❑ בכלל לא=4
❑ לא יודע=5 attitude2

לסוקר: קרא- בשאלות הבאות אשאל לגבי מצב בריאותך.

33. מה מצבך הבריאותי באופן כללי?

❑ טוב מאוד=1 ❑ טוב=2 ❑ לא כל כך טוב=3 ❑ לא טוב בכלל=4 health1

34. האם אי פעם רופא איבחן אצלך

❑ אנמיה מחוסר ברזל=1 ❑ אוסטיאופורוזיס, דלדול עצמות=2 ❑ כולסטרול גבוה=3
❑טריגליצרידים גבוהים=4 ❑ יתר לחץ דם=5 ❑ סוכרת, תלויה באנסולין, (לא סוכרת הריונית)=6 ❑ סוכרת, לא תלויה באנסולין (לא סוכרת הריונית)=7 ❑ שבץ מוחי=8
❑סרטן=9 health2

35. מה הגובה שלך (ללא נעליים)בס"מ? _____ מסרב/לא יודע/לא זוכר=n/a height

36. מתי נשקלת בפעם האחרונה? ❑ היום=1 ❑ השבוע=2 ❑ החודש=3 ❑ לפני יותר מחודש=4
weightime

37. מה משקלך (ללא נעליים, בבגדים קלים) בק"ג? _______ לא יודע/לא זוכר=n/a weight

38. האם אתה מעשן? ❑ כן=2 ❑ לא כיום, אבל עישנתי בעבר=1 ❑ לא, אף פעם לא עישנתי=0
smoking

אנחנו מעוניינים לבדוק באופן עקרוני עמדות ביחס לנכונות להשתתף במחקרי תזונה ובריאות. מה היא עמדתך לגבי הסכמתה להשתתף במחקר שבו את/ה תתבקשו לאשר גישה למידע רפואי אישי הקיים בתיקך בקופת החולים (מדידות משקל, תוצאות בדיקות דם וכד')? שאלה זו לא מתיחסת למחקר הנוכחי ולא נבקש כאן כל גישה לתיק רפואי.

25. האם תהיה/תהיי מוכנה באופן עקרוני להשתתף? ❑ כן=1 ❑ לא=0 future

תודה רבה על השתתפותך במחקר. כעת תוכל לקבל פיצוי כספי של 100 ₪ בתלושים עבור הזמן שהקדשת למחקר. על מנת לקבל את הפיצוי עליך להגיע לעמותת ______.

סוקר: מלא את כל הפרטים בסיום הראיון.

שעת סיום _______ timeend

האם היו קשיי שפה בראיון? ❑ כן=1 ❑ לא=0 langdif

האם היו קשיי הבנה בראיון? ❑ כן=1 ❑ לא=0 compdif

האם היו קשיי היענות/ שיתוף פעולה? ❑ כן=1 ❑ לא=0 cooper

האם השאלון נענה במלואו? ❑כן=1 ❑ לא=0 fullansw

במידה ולא, מדוע?____ fullansw_why

הערות נוספות- ____ comments

Food Frequency Questionnaire

בדוק שבכל שורה סימנת תכיפות -

אם גודל מנה או תמונה מודגשים בקו תחתון יש להקיף בעיגול את גודל המנה או התמונה שבחרת

מס מזון | תאור מזון | גודל מנה או תמונה | פחות מפעם בחודש | תכיפות אכילה ממוצעת בשנה שעברה

בחודש 1-3 | בשבוע 6-5 4-3 1 -2- | ביום +6 5-4 3-2 1

ביצים , חלב ומוצרי חלב

מס מזון	תאור מזון	גודל מנה או תמונה
1	מתכוני ביצים כגון חביתה, שקשוקה, עין	1 ביצה
2	ביצה קשה,רכה	1 ביצה
3	חלב 3 % שומן (כולל בקפה)	1/2 כוס
4	חלב 1 % שומן (כולל בקפה)	1/2 כוס
5	שוקו/משקה קקאו מוכן	1 כוס
6	גבינה לבנה או קוטג' 5 % - 9	1-כף או
6		חצי מיכל של 250 ג'
7	גבינה לבנה או קוטג' דל שומן 0.5 - 3 %	1-כף או
7		חצי מיכל של 250 ג'
8	שמנת חמוצה	גביע
9	יוגורט, לבן, אשל 4.5-3 % ללא תוספות ו/או ללא סוכר	גביע
10	יוגורט, לבן 1.5 % - 0 כולל רויון ללא תוספות	מיכל/כוס
11	מעדן חלב או גבינה, יוגורט, לבן 4.5 - 3 % עם תוספות ו/או סוכר	גביע
12	מעדן חלב או גבינה, יוגורט, לבן % 1 - 0 עם תוספות ו/או סוכר	גביע
13	גבינה צהובה (כולל בטוסט, פיצה , כל הסוגים)	1 פרוסה
14	גבינה מלוחה מכל סוג כולל צפתית, בולגרית, ברינזה (הקף תמונה)	תמונה 1,2,3

שומנים (כולל ברוטבים)

מס מזון	תאור מזון	גודל מנה או תמונה
15	חמאה	1 כפית
16	מרגרינה בנייר	1 כפית
17	מרגרינה רכה בגביע	1 כפית
18	מרגרינה לייט	1 כפית

עוף בשר דגים

מס מזון	תאור מזון	גודל מנה או תמונה
19	שניצל עוף או הודו - מתכון ביתי (הקף תמונה)	תמונה 4,5,6

בדוק שבכל שורה סימנת תכיפות -

אם גודל מנה או תמונה מודגשים בקו תחתון יש להקיף בעיגול את גודל המנה או התמונה שבחרת

מס מזון	תאור מזון	גודל מנה או תמונה	פחות מפעם בחודש	תכיפות אכילה ממוצעת בשנה שעברה							
				בחודש	בשבוע			-2 ביום			
				1-3	6-5	4-3	1	+6	5-4	3-2	1
20	מנת עוף או הודו עם עור	1/4 עוף או מנת הודו בינונית									
21	מנת עוף או הודו ללא עור	1/4 עוף או מנת הודו בינונית									
22	נקניקיות מכל סוג	1 יחידה									
23	נקניק כגון סלאמי (לא כולל פסטרמה)	1 פרוסה									
24	פסטרמה או חזה הודו מעושן	1 פרוסה									
25	קציצות הודו, בקר, עוף -מתכון ביתי	2 קציצות בינוניות									
26	שישליק, שווארמה בפיתה	1 מנה									
27	פלאפל בפיתה	1 מנה									
28	מזון בשרי קנוי מוכן-שניצל מצופה, קציצות, כולל המבורגר נאגט	1 מנה או 2 קציצות									
29	מוצרים צמחיים כולל המבורגר או שניצל סויה, קציץ סויה	1 יחידה									
30	בשר בלבד (בקר, עגל ,כבש, חזיר) כגון סטייק או גולש (הקף תמונה)	תמונה 12 11 10									
31	תבשילי בשר בקר מעורבים כגון מוסקה, קובה, ממולאים, חמין עם בשר	מנה בינונית									
32	תבשילי עוף או הודו מעורבים כגון חמין עם עוף, ממולאים עם עוף	מנה בינונית									
33	מרק בשר או עוף	קערת מרק									
34	אברים פנימיים-כבד, קורקבנים, לשון, לבבות (עוף, בקר,הודו) (הקף תמונה)	תמונה 9 8 7									
35	דג מבושל, אפוי או גריל (הקף תמונה)	תמונה 15 14 13									
36	דג מטוגן (הקף תמונה)	תמונה 15 14 13									
37	טונה משומר, סלט טונה	1 כף גדושה									
38	דג כבוש, יבש, מעושן או משומר לא כולל טונה	יחידה									

לחם ומוצרי מאפה

מס מזון	תאור מזון	גודל מנה או תמונה	פחות מפעם בחודש	1-3	6-5	4-3	1	+6	5-4	3-2	1
40	לחם או חלה רגילים (הקף תמונה)	תמונה 18 17 16									
41	לחם קל, חלה קלה או לחמניות קלות כולל לחמים קלים מלאים	1 פרוסה או חצי לחמניה									
42	לחם מקמח מלא או שיפון (לא קל) (הקף תמונה)	תמונה 18 17 16									
43	בגט	1/4 בגט ארוך									

בדוק שבכל שורה סימנת תכיפות -

אם גודל מנה או תמונה מודגשים בקו תחתון יש להקיף בעיגול את גודל המנה או התמונה שבחרת

מס מזון	תאור מזון	גודל מנה או תמונה		פחות מפעם בחודש	תכיפות אכילה ממוצעת בשנה שעברה בחודש 1-3	בשבוע 6-5	בשבוע 4-3	בשבוע 2-1	ביום +6	ביום 5-4	ביום 3-2	ביום 1
44	לחמניות רגילות או בייגל	1 יחידה										
45	פיתה	1 פיתה										
46	קרקרים מלוחים או מצה	3 קרקרים או חצי מצה										
47	בורקס	1 קטן										
48	ג'חנון, מלוואח, קובנה, סיגרים	יחידה										
49	פיצה	משולש										
	**דגנים**											
50	דגני בוקר כגון קורנפלקס או גראנולה (הקף תמונה)	תמונה	19 20 21									
51	דגני בוקר מבושלים כגון דייסת קוואקר, סולת	קערה										
	**עמילנים וקטניות**											
52	אורז לבן וצהוב עם או בלי תוספות (הקף תמונה)	תמונה	22 23 24									
53	קוסקוס, כוסמת, בורגול, ממליגה גריסים מבושלים (הקף תמונה)	תמונה	22 23 24									
54	תפוחי אדמה מבושלים, אפויים, פירה, סלט תפוחי אדמה (הקף תמונה)	תמונה	28 29 30									
55	צ'יפס (לא כולל חטיף) (הקף תמונה)	תמונה	31 32 33									
56	פסטה, פתיתים מכל סוג (הקף תמונה)	תמונה	34 35 36									
57	קטניות מבושלות , כולל גרגרי חומוס, שעועית, גרגרי סויה	1/2 כוס										
	**פירות-כפי שנאכלים בעונה**											
58	מנדרינה או קלמנטינה	1 בינוני										
59	תפוז או ½ אשכולית	1 בינוני										
60	מיץ תפוזים או אשכוליות טבעי או קפוא	1 כוס										
61	תפוח עץ - כל הצורות	1 בינוני										
62	משמש טרי או יבש או שסק	2 משמשים או 1 שסק										
63	ענבים או צימוקים	מנה = 12 יחידות										
64	בננה	1 בינונית										

בדוק שבכל שורה סימנת תכיפות -

אם גודל מנה או תמונה מודגשים בקו תחתון יש להקיף בעיגול את גודל המנה או התמונה שבחרת

מס מזון	תאור מזון	גודל מנה או תמונה		פחות מפעם בחודש	תכיפות אכילה ממוצעת בשנה שעברה בחודש 1-3	בשבוע 6-5	בשבוע 4-3	בשבוע 2- 1	ביום +6	ביום 5-4	ביום 3-2	ביום 1
65	מלון	1/4 מלון										
66	קיווי (יחידה) או תות שדה (1 כוס)	1 יחידה										
67	מנגו	1 בינוני										
68	אפרסק, נקטרינה, שזיף (כולל יבש)	1 בינוני										
69	אגס טרי , מבושל או משומר	1 בינוני										
70	אפרסמון	1 בינוני										
71	אבטיח	1/8 אבטיח										
72	שאר פירות יבשים (כולל תמרים, תאנים ואחרים)	1 יחידה										
73	סלט פירות , קוקטייל או ליפתן פירות מבושלים, דובדבנים רימונים ליצי	1/2 כוס										
	**ירקות**											
74	עגבנייה טרייה או ½ כוס עגבניות שרי	1 יחידה										
75	עגבניות מבושלות כולל רוטב עגבניות או מרק עגבניות	1/2 כוס										
76	פלפל אדום	1 פלפל										
77	פלפל ירוק	1 פלפל										
78	מלפפון או סלט מלפפונים	1 מלפפון										
79	קישואים או חצילים (הקף תמונה)	תמונה	37 38 39									
80	אפונה, שעועית ירוקה או במיה מבושלת (הקף תמונה)	תמונה	37 38 39									
81	כרובית או ברוקולי	1/2 כוס										
82	בטטה מתוקה (הקף תמונה)	תמונה	28 29 30									
83	כרוב ירוק או אדום כולל כרוב ניצנים (הקף תמונה)	תמונה	40 41 42									
84	חסה טרייה כולל סלט חסה (הקף תמונה)	תמונה	40 41 42									
85	גזר, טרי או מבושל, כולל מיץ גזר	1 גזר או 1/4 כוס מיץ										
86	תירס	קלח										
87	פטרוזיליה, סלרי, שמיר, כוסברה, בצל ירוק	כף ירק קצוץ										

בדוק שבכל שורה סימנת תכיפות -

אם גודל מנה או תמונה מודגשים בקו תחתון יש להקיף בעיגול את גודל המנה או התמונה שבחרת

תכיפות אכילה ממוצעת בשנה שעברה -2-

מס מזון	תאור מזון	גודל מנה או תמונה		פחות מפעם בחודש	בחודש 1-3	בשבוע 6-5	בשבוע 4-3	בשבוע 1	ביום +6	ביום 5-4	ביום 3-2	ביום 1
88	סלט טרי ללא רוטב או שמן (לא כולל מה שציינת בנפרד עד כה)(הקף	תמונה	40 41 42									
89	סלט טרי עם רוטב או שמן (לא כולל מה שציינת בנפרד עד כה) הקף תמונה)	תמונה	40 41 42									
90	אבוקדו, כולל סלט אבוקדו	1 כף גדושה										
91	לימון	1/2 לימון										
92	בצל	1/4 בצל										
93	שום	שן שום										
94	מרק ירקות או תוספת ירקות מעורבים	1 קערה										
95	סלט חומוס	1 כף גדושה										
96	סלט טחינה כולל רוטב טחינה	1 כף גדושה										
97	סלטי ירקות קנויים מעובדים כגון - חצילים, טורקי, מטבוחה, כרוב	1 כף גדושה										
98	ירקות מוחמצים, חמוצים, לא זיתים	יחידה										
99	זיתים	10 זיתים										

חטיפים ועוגות

מס מזון	תאור מזון	גודל מנה או תמונה		פחות מפעם בחודש	בחודש 1-3	בשבוע 6-5	בשבוע 4-3	בשבוע 1	ביום +6	ביום 5-4	ביום 3-2	ביום 1
100	שוקולד חלב או מריר או חטיפי שוקולד מכל סוג	6 קוביות או 30 גר'										
101	חטיפים מלוחים כגון ביסלי, צ'יפס, בייגלה, שקדי מרק	חופן או 25 גר'										
102	עוגות גבינה או עוגות קרם (הקף תמונה)	תמונה	43 44 45									
103	עוגות ועוגיות שמרים כגון רוגאלך, קרואסון או סופגניה	1 יחידה או פרוסה										
104	עוגות תופין או טורט כולל עוגת שוקולד (הקף תמונה)	תמונה	43 44 45									
105	עוגת פירות כולל פאי פירות (הקף תמונה)	תמונה	43 44 45									
106	וופלים ועוגיות ממולאות או מצופות	1 יחידה										
107	עוגיות פשוטות כולל ביסקוויטים ועוגיות ללא ציפוי	1 יחידה										
108	גלידה או ארטיק	1 ארטיק או 2 כדורים										
109	גרעינים שחורים או לבנים כולל גרעיני אבטיח	חופן או 25 גר'										
110	אגוזים, שקדים, פיסטוקים	חופן או 25 גר'										

בדוק שבכל שורה סימנת תכיפות -

אם גודל מנה או תמונה מודגשים בקו תחתון יש להקיף בעיגול את גודל המנה או התמונה שבחרת

מס מזון	תאור מזון	גודל מנה או תמונה	פחות מפעם בחודש	בחודש 1-3	בשבוע 1	בשבוע 4-3	בשבוע 6-5	ביום +6	ביום 5-4	ביום 3-2	ביום 1
111	בוטנים	חופן או 25 גר'									
112	שמן כתוספת לסלטים, תבשילים וכו'	1 כפית									
113	מיונז, כולל קל	1 כפית									
114	דבש, ריבה, סירופ פרי, או סירופ מיפל	1 כפית									
115	סוכר	1 כפית									
116	ממתיקים מלאכותיים כגון סוכרזית, מתוק וקל	1 יחידה									

משקאות

מס מזון	תאור מזון	גודל מנה או תמונה									
117	נקטר מכל סוג כולל סיידר	1 כוס									
118	מיץ בטעם פרי כולל משקה מיץ, סירופ מוכן מדולל	1 כוס									
119	משקאות דיאט מכל סוג	1 כוס									
120	משקאות תוססים רגילים (עם סוכר) מכל סוג	1 כוס									
121	קפה ללא קופאין	1 ספל									
122	קפה ונס קפה	1 ספל									
123	תה צמחים	1 ספל									
124	תה רגיל	1 ספל									
125	בירה	פחית או 1.5 כוסות									
126	יין יבש מתוק קוקטלים	1 כוסית									
127	משקאות אלכהולים חריפים	1 כוסית									

Food Security and Leket Israel Survey Codebook

שם הסוקר: __________ intname

اسم الباحث:____________

تم عقد المقابلة بتاريخ:__________ ساعة البداية:_________

رقم مجري الأستبيان________ جمعية___________

في حالة اذا تم تأجيل التقرير:تاريخ المواصلة _________ ساعة البدء_________ ساعة الانتهاء:

تاريخ المواصلة _________ ساعة البدء_________ ساعة الانتهاء:

للباحث :أقرأ-"مرحبا , اسمي_______ وأني أتصل لكي أدعو حول المشاركة في البحث حول موضوع الأمن الغذائي. هل وافقت ان يتم الاتصال بك اليوم بهذه الساعة, أنستطيع مواصلة المقابلة؟ "

نعم- نواصل . كلا--- هل يمكننا ان نعقد المقابلة في وقت أخر؟ نعم, في أي وقت ؟ سجل المعلومات مع تفاصيل مجري الاتصال .

في حالة تم تأجيل التقرير : متى طلب الاستمرار؟______________________

للباحث: اقرأ: قبل ان نبدأ الاستبيان, حصلت على اوراق من الجمعية والتي تحتوي على صور لمأكولات مختلفة, هل تلك الاوراق موجودات معك الآن؟

❑ نعم =1 نكمل الاستبيان

❑ كلا=0 (للباحث أقرأ) هل ليك هاتف خليوي ذكي؟ استطيع ارسال الصور عن طريق رسالة لهاتفك, هل انت معنيٌ بذلك؟ نستطيع الانتظار حتى تستلم الاوراق من الجمعية في حالة عدم موافقتك على ارسال الصور لهاتفك الخليوي.

❑ نعم =1 ❑ كلا=0 (نشكر المشترك بلباقة على المشاركة ونطلب منه الانتظار حتى استلام الاوراق من الجمعية).

** اذا وافق المشترك على ارسال الصور له الكترونيا, يجب ارسال الصور لرقم هاتفه, ويجب التأكد ان الرسالة وصلت لهاتفه وباستطاعته رؤية الصور بشكل واضح**

للباحث: يجب القول: "حاليا نكمل الاستبيان , وعند الحاجة للصور, اطلب منك النظر الى الاوراق التي بحوزتك"

للباحث أقرأ-"أود أن اذكرك ان هذا الاستبيان من دون ذكر أسماء وهو جزء من البحث. عدم الموافقة بالمشاركة سوف تنعكس بعدم استكمال الأستبيان . تستطيع الأجابة على جزء من الاسئلة فقط. تستطيع/تستطيعين في أي وقت ان توقف/توقفي مشاركتك في البحث, من دون المس بحقوقك, من دون المس بك,ومن دون أي عقوبة, بما في ذلك الحق في الحصول على المساعدة من الجمعية. في حالة عدم شعورك بالراحة , الرجاء اخباري ".

1.هل توافق /توافقين على المواصلة بالمقابلة؟ ❑ نعم =1 ❑ كلا=0 consent

للباحث- بعد أن تجلس وتستعد لبدء المقابلة - اقرأ: "من الممكن أن لا يكون لديك خبرة بهذا النوع من الاستبيانات لذا أود ان أشرحها لك.سوف أقرا لك الاسئلة كما هي مكتوبة, وسوف اطلب منك الاجابة باحدى الطريقتين: بجزء من الاسئلة تستطيع اعطاء أجوبة مفتوحة

وبجزء تقوم باختيار واحد او اكثر من بين الاختيارات التي سوف اقرؤها لك. اذا واجهت صعوبة أو عدم وضوح في أي سؤال اخبرني رجاءا."

2 .في اي سنة ولدت ؟__________birthyear

3.الجنس : ❑ انثى=1 ❑ذكر=2 sex

تفاصّل ديموغرافية

للباحث: اقرأ- "سوف أتطرق الان لأسئلة عامة عن نفسك وعن عائلتك ".

4.كم عمرك؟ ______ (للباحث: تأكد أنه يطابق تاريخ الميلاد)age

5.أين ولدت:____________________country

6.فًي أي سنة هاجرت إلى اسرائيل؟___________ immigyear

7.الحالة الشخصية؟

❑أعزب \ عزباء= 1 ❑ متزوج \ متزوجة =2 ❑مطلق \ مطلقة =3 ❑أرمل \ أرملة=4 ❑منفصل \ منفصلة=5

غير ذلك, حدد_______

8.هل أنت:

❑يهودي =1 ❑عربي مسلم =2 ❑ عربي مسّيحًي= 3 ❑ درزي =4 ❑ مسّيحًي = 5

غًير ذلك، حدد __________

9.خلال الثلاث أشهر الماضّية، هل أنت:

❑عاطل عن العمل بسبب مرض / عجز / اعاقة =1 ❑تعمل كأجير =2 ❑فًي اجازة ولادة =3

❑ تعمل كمستقل= 4 ❑متقاعد=5 ❑طالب يعمل= 6 ❑عاطل عن العمل = 7

❑عامل مع العائلة غير مدفوع الاجر=8 ❑مدبرة منزل =9 ❑طالب (لا تعمل) =10 ❑عضو في مستوطنة /كيبوتس=11 ❑لا تعمل لاسباب أخرى=12 غًير ذلك، حدد _________

10.ما هو دخلك الشهري الصافًي من العمل؟___________________ لاتعرف/ترفض /ي الاجابة =n/a income

11.كم شخص يسكن فًي المنزل بشكل ثابت (لا يشمل مستأجر، جندي فًي فترة الخدمة العسكرية، طالب يسكن فًي دور طلبة أو خارج المنزل)؟ ____________ .

12.اذكر عدد الأشخاص الذيّن يسكنون المنزل وعلاقتك بكل واحد منهم, جنسهم وجيلهم. على سبيل المثال: زوج/زوجة , أب/أم , جد/جدّة , أبن/أبنة , أخ/أخت, صديق/رفيق, غير ذلك (بالقائمة أدناه)

العدد (لايشمل المشترك)	صلة القرابة	الجنس	الجيل
2	hh2	hh2_sex	hh2_age
3	hh3	hh3_sex	hh3_ age
4	hh4	hh4_sex	hh4_ age
5	hh5	hh5_sex	hh5_ age
6	hh6	hh6_sex	hh6_ age
7	hh7	hh7_sex	hh7_ age
8	hh8	hh8_sex	hh8_ age
9	hh9	hh9_sex	hh9_ age
10	hh10	hh10_sex	hh10_ age

❑زوج/زوجة =1 ❑ أب/أم =2 ❑ جد/جدّة=3 ❑ أبن/أبنة=4 ❑أخ/أخت =5 ❑
صديق/رفيق =6 ❑غير ذلك =7

للباحث :أقرأ-" الان سوف أطلب منك تفاصيل عن الطعام الذي تستهلكه . سوف أقرأ قائمة من المنتوجات الغذائية ولكل منتوج سوف أسألك كم مرة تستهلك هذا المنتوج ."

(עבור לשאלון FFQ) (أنتقل لأستبيان FFQ)

أستبيان الأمن الغذائي_ لاحظ أذا ما الأسئلة ملائمة للعائلات مع أطفال او من دون(الاسئلة 16-18,26-30 مخصصة فقط للعائلات مع أطفال):

للباحث – أقرأ: "سوف أقرأ لك عدد من الجمل التي قالوها أشخاص حول الغذاء المتوفر في البيت . ولكل جملة سوف تقول لي هل بالسنة الأخيرة , هذه الجملة كانت عالأغلب صحيحة, صحيحة بشكل ما, أو غير صحيحة بتاتا بالنسبة لك أو لأفراد عائلتك ."

13."خشينا من انتهاء الطعام قبل ان يكون بحوزتنا نقود (مال) لنشتري المزيد."

❑عالأغلب صحيحة=1 ❑ صحيحة بشكل ما =2 ❑ غير صحيحة بتاتا =3 ❑ لا أعرف=4

❑أرفض الأجابة=5

14."الطعام الذي أشتريناه لم يكن كاف, ولم يكن بحوزتنا نقود لنشتري المزيد."

❑عالأغلب صحيحة=1 ❑ صحيحة بشكل ما =2 ❑ غير صحيحة بتاتا=3 ❑ لا أعرف=4

❑أرفض الأجابة=5

15." لم يكن بحوزتنا النقود الكافية لتناول وجبات متنوعة."

❑عالأغلب صحيحة=1 ❑ صحيحة بشكل ما =2 ❑ غير صحيحة بتاتا=3 ❑ لا أعرف=4

❑أرفض الأجابة=5

16." اعتمدنا على عدد من الأطعمة الغير مكلفة لإطعام الأطفال، لأن المال في حوزتنا قد انتهى" (سؤال للعائلات مع أطفال)

❑عالأغلب صحيحة=1 ❑ صحيحة بشكل ما =2 ❑ غير صحيحة بتاتا=3 ❑ لا أعرف=4

❑أرفض الأجابة=5

17."لم نتمكن من توفير وجبات متوازنة ومتنوعة للأطفال لأنه لم يكن في حوزتنا نقود" (سؤال للعائلات مع أطفال)

❑عالأغلب صحيحة=1 ❑ صحيحة بشكل ما =2 ❑ غير صحيحة بتاتا=3 ❑ لا أعرف=4

❑أرفض الأجابة=5

18."الأطفال لم يأكلوا بشكل كاف لأنه لم نتمكن من شراء الغذاء الكافي" (سؤال للعائلات مع أطفال)

❑عالأغلب صحيحة=1 ❑ صحيحة بشكل ما =2 ❑ غير صحيحة بتاتا=3 ❑ لا أعرف=4

❑أرفض الأجابة=5

19.في العام الماضي, هل أنت او اخرين في البيت قاموا بتقليل حجم وجباتهم, أو تخطوا وجبات الطعام لعدم وجود المال لشراء الطعام؟

❑ نعم=1 ❑ كلا=2 ❑ لا أعرف=3 ❑ أرفض الأجابة=4

20.كم مرة حصل ذلك ؟

❑تقريبا كل شهر=1 ❑ اشهر نعم, اشهر لا=2 ❑ فقط شهر واحد أو شهرين خلال السنة=3

❑لا أعرف=4 ❑ أرفض الأجابة=5

21." هل في العام الماضي أكلتم أقل مما أردتم أن تأكلوا, لانه لم يكن بحوزتكم المال الكافي لشراء الغذاء ؟

❑ نعم=1 ❑ كلا=2 ❑ لا أعرف=3 ❑ أرفض الأجابة=4

22.هل في العام الماضي انت والاخرين في البيت شعرتم بالجوع ولم تأكلوا لأنه لم يكن بحوزتكم المال الكافي لشراء الغذاء ؟

❑ نعم=1 ❑ كلا=2 ❑ لا أعرف=3 ❑ أرفض الأجابة=4

23.هل في العام الماضي انت والاخرين في البيت أنخفض وزنكم لانه لم يكن بحوزتكم المال الكافي لشراء الغذاء ؟

❑ نعم=1 ❑ كلا=2 ❑ لا أعرف=3 ❑ أرفض الأجابة=4

24.خلال العام الماضي, هل أنت والاخرين في البيت لم تأكلوا لمدة يوم كامل لانه لم يكن بحوزتكم المال الكافي لشراء الغذاء؟

❑ نعم=1 ❑ كلا=2 ❑ لا أعرف=3 ❑ أرفض الأجابة=4

25.كم مرة حصل ذلك؟

❑تقريبا كل شهر=1 ❑ اشهر نعم, اشهر لا=2 ❑ فقط شهر واحد أو شهرين خلال السنة=3
❑لا أعرف=4 ❑ أرفض الأجابة=5

26.هل في العام الماضي قللتم من حجم وجبات الطعام لطفلك/ اطفالك لأنه لم يكن هناك ما يكفي من المال لشراء الغذاء؟(سؤال للعائلات مع أطفال)

❑ نعم=1 ❑ كلا=2 ❑ لا أعرف=3 ❑ أرفض الأجابة=4

27.هل في العام الماضي، تخطى الأطفال وجبات الطعام لأنه لم يكن هناك ما يكفي من المال لشراء الغذاء؟(سؤال للعائلات مع أطفال)

❑ نعم=1 ❑ كلا=2 ❑ لا أعرف=3 ❑ أرفض الأجابة=4

28.كم مرة هذا يحصل ؟(سؤال للعائلات مع أطفال)

❑تقريبا كل شهر=1 ❑ اشهر نعم, اشهر لا=2 ❑ فقط شهر واحد أو شهرين خلال السنة=3
❑لا أعرف=4 ❑ أرفض الأجابة=5

29.هل في العام الماضي طفلك/ اطفالك كان/كانوا جائع/جائعين ولم يكن بأمكانك شراء المزيد من الغذاء ؟(سؤال للعائلات مع اطفال)

❑ نعم=1 ❑ كلا=2 ❑ لا أعرف=3 ❑ أرفض الأجابة=4

30.خلال العام الماضي, هل الأطفال في البيت لم يأكلوا لمدة يوم كامل لانه لم يكن بحوزتكم المال الكافي لشراء الغذاء؟(سؤال للعائلات مع أبناء)

❑ نعم=1 ❑ كلا=2 ❑ لا أعرف=3 ❑ أرفض الأجابة=4

الباحث : أقرأ-"الان سوف اسالك عن رايك بمواضيع تتعلق بالتغذية ."

31.الى أي مدى انت مهتم بالعلاقة بين التغذية و الصحة؟

❑ إلى مدى كبير جدا =1 ❑ إلى مدى كبير= 2 ❑ إلى مدى قليل =3 ❑ لا اهتم اطلاقا=4
❑ لا أعرف =5

32.الى أي مدى تتأثر عاداتك الغذائية بالمعلومات او الدعايات عن العلاقة بين التغذية والصحة؟

❑ إلى مدى كبير جدا =1 ❑ إلى مدى كبير= 2 ❑ إلى مدى قليل =3 ❑ لا اهتم اطلاقا=4
❑ لا أعرف =5

للباحث : أقرأ- " في الاسئلة التالية سوف أسألك عن وضعك الصحي ".

33.ماهو وضعك الصحي بشكل عام؟

❑ جيد جدا= 1 ❑ جيد= 2 ❑ ليس جيدا إلى هذا الحد =3 ❑ ليس جيدا اطلاقا= 4

34.هل حدث وقال لك طبيب انه لديك:

❑فقر دم بسبب نقص الحديد=1 ❑ هشاشة عظام (اوستيوبوروسيس), ترقق عظام=2 ❑كولسترول عالٍ=3
❑دهنيات ثلاثية عالية =4 ❑ ضغط دم عالٍ=5 ❑ سكري معتمد على الأنسولين، (لا يشمل سكري الحمل)=6 ❑سكري غير معتمد على الأنسولين، (لا يشمل سكري الحمل)=7 ❑ جلطة دماغيّة=8
❑سرطان=9

35.ما هو طولك (بدون حذاء) بالسم؟ __________أرفض الأجابة/ لاأعرف/لاأذكر =n/a height

36.متى قست وزنك في الفترة الاخيرة ؟ ❑اليوم =1 ❑هذا الاسبوع =2 ❑هذا الشهر= 3 ❑ قبل أكثر من شهر= 4
weightime

37.ما هو وزنك (بدون حذاء, بملابس خفيفة)بكغم؟___________ لاأعرف/لاأذكر= n/a weight

38.هل تدخن؟ ❑ نعم=2 ❑ لا، ولكن دخنت في السابق=1 ❑ لا، لم أدخن أبدا=0 smoking

هذا السؤال لا يتعلق بالبحث الحالي .نريد أن نعرف مدى موافقتك المبدئية في المشاركة في ابحاث تتعلق في مجال التغذية والصحة. ما هو موقفك من المشاركة في ابحاث تتطرق الى الحصول على المعلومات الطبية الشخصية الموجودة في سجلك الخاص في صندوق المرضى(قياسات الوزن، واختبارات الدم، الخ)؟وهل توافق على المشاركة في ابحاث تتطلب منك معلومات طبية في سجلك الطبي.هذا السؤال لا يتعلق بالبحث الحالي ولن نطلب الوصول إلى السجلات الطبية في هذا البحث.

25.هل ستكن مستعد /مستعدة للمشاركة بشكل مبدئي؟ ❑ **نعم=1** ❑ **كلا=0** future

شكرا جزيلا لك على المشاركة في البحث. الآن يمكنك الحصول على تعويض مالي 100 ₪ بكوبونات الجمعية كتعويض على الوقت الذي خصصته للبحث. من أجل الحصول على تعويضات تحتاج الوصول الى جمعية_________-

סוקר: מלא את כל הפרטים בסיום הראיון.

שעת סיום _______ timeend

האם היו קשיי שפה בראיון? ❑ כן=1 ❑ לא=0 langdif

האם היו קשיי הבנה בראיון? ❑ כן=1 ❑ לא=0 compdif

האם היו קשיי היענות /שיתוף פעולה? ❑ כן=1 ❑ לא=0 cooper

האם השאלון נענה במלואו? ❑כן=1 ❑ לא=0 fullansw

במידה ולא, מדוע?____ fullansw_why

הערות נוספות- ____ comments

סיכום רכזת השאלון

הגיע בתאריך .... בשעה.......:..

הוקלד ע"י..............בתאריך: ....:

נבדק ....:

דורש תיקון כן/לא

תיקוני הסוקר הוחזרו בתאריך: __________

תיקונים נוספים: ________________

השאלון הוקלד סופית בתאריך:____ ע"י: ______

סיום טיפול: כן/לא

تحقق أذا تمت الاشارة في كل سطر على الوتيرة-

أذا حجم الوجبة أو الصورة مشار أليها بخط أحط بدائرة حجم

**رقم المنتوج الغذائي**	**وصف الغذاء**	**حجم الوجبة / صورة**	**بأي وتيرة تم أستهلاك المنتوج الغذائي بالمعدل خلال العام الماضي**								
			اقل من مرة في الشهر	1-3مرات في الشهر	1-2 مرات فًي الاسبوع	3-4 مرات فًي الاسبوع	5-6مرات فًي الاسبوع	مرة باليوم	2-3 مرات باليوم	4-5 مرات باليوم	6مرات باليوم عالأقل
1	وصفات طعام من البيض مثل: عجة, شقشوقة , بيضة عين	بيضة واحدة									
2	بيضة مسلوقة , برشت	بيضة واحدة									
3	حليب 3% دهون (يشمل الحليب في القهوة)	1/2 كأس									
4	حليب 1% دهون (يشمل الحليب في القهوة)	1/2 كأس									
5	شوكو /مشروب كاكاو جاهز التحضير	كأس									
6	جبنة بيضاء أو كوتيتج 5-9%	ملعقة-أو نصف علبة ذات وزن 250غرام									
7	جبنة بيضاء أو كوتيتج قليل الدسم 0.5-3%	ملعقة-أو نصف علبة ذات وزن 250غرام									
8	قشدة (شمينت) حامضة	عبوة واحدة									
9	زبادي (يوجورت), لبن,ايشيل3-4.5% من دون أي أضافات و/أو من دون سكر .	عبوة واحدة									
10	زبادي (يوجورت), لبن0-1.5% بما في ذلك القشطة من دون أي أضافات.	كأس									
11	منتوج حليب أو جبنة , يوجورت, لبن3-4.5%يحوي أضافات و/أو سكر	عبوة واحدة									
12	منتوج حليب أو جبنة , يوجورت, لبن 0-1% يحوي أضافات و/أو سكر	عبوة واحدة									
13	جبنة صفراء(بما في ذلك الجبنة التي في التوست , بالبيتسا,كل الانواع)	قطعة واحدة									
14	جبنة مالحة بكل الأنواع الجبنة الفرنسية،البلغارية، برينز (أحط بدائرة الصورة الملائمة)	صورة 1,2,3									

تحقق أذا تمت الاشارة في كل سطر على الوتيرة-

أذا حجم الوجبة أو الصورة مشار أليها بخط أحط بدائرة حجم

الدهنيات (بما في ذلك الدهنيات المتواجدة في الصلصات)

رقم المنتوج الغذائي	وصف الغذاء	حجم الوجبة / صورة	بأي وتيرة تم أستهلاك المنتوج الغذائي بالمعدل خلال العام الماضي								
			اقل من مرة في الشهر	1- 3مرات في الشهر	1-2 مرات فًي الاسبوع	3-4 مرات فًي الاسبوع	5- 6مرات فًي الاسبوع	مرة باليوم	2-3 مرات باليوم	4-5 مرات باليوم	عالأقل 6مرات باليوم
15	زبدة	ملعقة صغيرة									
16	مرجرين (بالورق)	ملعقة صغيرة									
17	مرجرين لين /سائل (في عبوة)	ملعقة صغيرة									
18	مرجرين لايت	ملعقة صغيرة									

دجاج,لحوم,أسماك

رقم المنتوج الغذائي	وصف الغذاء	حجم الوجبة / صورة	بأي وتيرة تم أستهلاك المنتوج الغذائي بالمعدل خلال العام الماضي								
			اقل من مرة في الشهر	1- 3مرات في الشهر	1-2 مرات فًي الاسبوع	3-4 مرات فًي الاسبوع	5- 6مرات فًي الاسبوع	مرة باليوم	2-3 مرات باليوم	4-5 مرات باليوم	عالأقل 6مرات باليوم
19	شنيتسل دجاج أو حبش _ صنع البيت (أحط بدائرة الصورة الملائمة)	صورة 4,5,6									
20	وجبة دجاج أو حبش من دون نزع الجلد	1/4 دجاجة أو وجبة حبش متوسطة									
21	وجبة دجاج أو حبش منزوع الجلد	1/4 دجاجة أو وجبة حبش متوسطة									
22	نقانق (كل الأنواع)	قطعة / وحدة									
23	مرتديلا , مثل: مرتديلا سلامي (لا يشمل البسطرامة)	شريحة واحدة									
24	بسطرامة أو صدر حبش مدخن	شريحة واحدة									
25	كرات الحبش,اللحم, الدجاج_ صنع البيت	2 كرات متوسطة									
26	رغيف مشاوي (شيشليك), شاورمة	وجبة واحدة									
27	رغيف فلافل	وجبة واحدة									
28	طعام لحمي- جاهز التحضير قد تم شرائه مثل: هامبرجر شنيتسل ,كرات اللحم,	وجبة واحدة أو2 كرات متوسطة									
29	منتوجات نباتية مثل: شنيتسل أو هامبرجر المصنوع من الصويا, كرات الصويا	قطعة / قسم واحد									
30	لحمة (عجل,خروف ,بقر,خنزير) مثل ستيك /كفتة(جولش) (أحط بدائرة الصورة الملائمة)	صورة 10,11,12									
31	وصفات طبخ باللحم مثل : كبة, يخنة باللحم ,محاشي	وجبة متوسطة									
32	وصفات طبخ بالدجاج أو الحبش مثل : يخنة بالدجاج ,محاشي بالدجاج	وجبة متوسطة									
33	شوربة لحم أو دجاج	كأس شوربة واحدة									
34	أعضاء داخلية مثل : كبد,لسان,قلوب(دجاج,حبش,خروف,ع	صورة 7,8,9									

	جل) (أحط بدائرة الصورة الملائمة)										
35	سمك مطبوخ مشوي بالفرن أو على فحم (أحط بدائرة الصورة الملائمة)	صورة 13,14,15									
36	سمك مقلي (أحط بدائرة الصورة الملائمة)	صورة 13,14,15									
37	تونة محفوظة, سلطة تونة	1 ملعقة كبيرة (معرمة)									
38	سمك محفوظ,مدخن مجفف,مكبوس لايشمل التونة	قطعة / قسم واحد									

الخبز ومنتوجاته

رقم المنتوج الغذائي	وصف الغذاء	حجم الوجبة / صورة	بأي وتيرة تم أستهلاك المنتوج الغذائي بالمعدل خلال العام الماضي								
			اقل من مرة في الشهر	-1 3مرات في الشهر	2-1 مرات في الاسبوع	4-3 مرات في الاسبوع	-5 6مرات في الاسبوع	مرة باليوم	3-2 مرات باليوم	5-4 مرات باليوم	عالأقل 6مرات باليوم
40	خبز (الأبيض)/ حالاه (أحط بدائرة الصورة الملائمة)	صورة 16,17,18									
41	خبز ,لخمنيوت الخفيف (الذي يحوي نخالة /قشرة القمح) بما في ذلك كل منتوجات الخبز الخفيف	قطعة/ شريحة واحدة أو 1/2 لخمنيوت									
42	خبز القمح الكامل أو شوفان (أحط بدائرة الصورة الملائمة)	صورة 16,17,18									
43	باجيت	1/4باجيت كبير									
44	لخمنيوت أو بييجل	قطعة واحدة									
45	رغيف خبز	رغيف واحد									
46	مصة أو كركيرمالح (بسكوت مالح)	1/2 مصة أو 3 قطع كركير									
47	بوريكس	1 صغير									
48	وصفات طعام بعجينة الرقائق	قطعة واحدة									
49	بيتسا	قطعة واحدة (مثلث)									

الحبوب

رقم المنتوج الغذائي	وصف الغذاء	حجم الوجبة / صورة	بأي وتيرة تم أستهلاك المنتوج الغذائي بالمعدل خلال العام الماضي								
			اقل من مرة في الشهر	-1 3مرات في الشهر	2-1 مرات فًي الاسبوع	4-3 مرات فًي الاسبوع	-5 6مرات فًي الاسبوع	مرة باليوم	3-2 مرات باليوم	5-4 مرات باليوم	عالأقل 6مرات باليوم
50	حبوب الصباح مثل: رقائق الصباح(كورنفلكس) /جرنولا (أحط بدائرة الصورة الملائمة)	صورة 19,20,21									
51	حبوب صباح مطبوخة مثل دايست كواكر/سميد	كأس شوربة									

تحقق أذا تمت الاشارة في كل سطر على الوتيرة-

أذا حجم الوجبة أو الصورة مشار أليها بخط أحط بدائرة حجم

النشويات والبقوليات

رقم المنتوج الغذائي	وصف الغذاء	حجم الوجبة / صورة	بأي وتيرة تم أستهلاك المنتوج الغذائي بالمعدل خلال العام الماضي								
			اقل من مرة في الشهر	1-3مرات في الشهر	1-2 مرات في الاسبوع	3-4 مرات في الاسبوع	5-6مرات في الاسبوع	مرة باليوم	2-3 مرات باليوم	4-5 مرات باليوم	عالأقل 6مرات باليوم
52	رز أبيض /أصفر مع أو بدون إضافات (أحط بدائرة الصورة الملائمة)	صورة 22,23,24									
53	كسكس,قمح,برغل ,فريكة مطبوخين (أحط بدائرة الصورة الملائمة)	صورة 22,23,24									
54	بطاطا مطبوخة,مشوية بالفرت,مهروسة,سلطة بطاطا(أحط بدائرة الصورة الملائمة)	صورة 28,29,30									
55	رقائق البطاطا (لا يشمل المسليات) (أحط بدائرة الصورة الملائمة)	صورة 31,32,33									
56	معكرونة (باستا) ,بتيتيم يشمل كل الأنواع (أحط بدائرة الصورة الملائمة)	صورة 34,35,36									
57	بقوليات مطبوخة , يشمل حبوب الحمص,الفاصوليا,حبوب الصويا	1/2 كأس									

الفاكهة—كما تؤكل في موسمها

رقم المنتوج الغذائي	وصف الغذاء	حجم الوجبة / صورة	بأي وتيرة تم أستهلاك المنتوج الغذائي بالمعدل خلال العام الماضي								
			اقل من مرة في الشهر	1-3مرات في الشهر	1-2 مرات في الاسبوع	3-4 مرات في الاسبوع	5-6مرات في الاسبوع	مرة باليوم	2-3 مرات باليوم	4-5 مرات باليوم	عالأقل 6مرات باليوم
58	مندالينا ,كلمنتينا	1 متوسطة									
59	برتقال أو 2/1 جريفروت	1 متوسطة									
60	عصير برتقال أو جريفروت طبيعي أو مجمد	1/2 كأس									
61	تفاح_بجميع الأشكال	1 متوسطة									
62	مشمش طازج أو مجفف أو أكيدنيا	2 حبات مشمش أو 1 أكيدنيا									
63	عنب أو زبيب	وجبة =12 حبة									
64	موز	1 متوسطة									
65	شمام	1/4 شمامة									
66	كيوي (قطعة)أو توت أرضي (كأس)	قطعة									
67	مانجا	1 متوسطة									
68	خوخ, خوخ أملس بروميا(يشمل المجفف)	1 متوسطة									
69	أجاص طازج,مطبوخ أو محفوظ	1 متوسط									
70	أفرسمون	1 متوسط									
71	بطيخ	1/8 بطيخة									

72	غيرها من الفاكهة المجففة (يشمل تمر,تين وغيره)	قطعة									
73	سلطة فاكهة , كوكتيل أو خلطة فاكهة مطبوخة ,كرز,رمان وليتشي	1/2 كأس									

الخضار

رقم المنتوج الغذائي	وصف الغذاء	حجم الوجبة / صورة	بأي وتيرة تم أستهلاك المنتوج الغذائي بالمعدل خلال العام الماضي								
			اقل من مرة في الشهر	1-3مرات في الشهر	1-2 مرات في الاسبوع	3-4 مرات في الاسبوع	5-6مرات في الاسبوع	مرة باليوم	2-3 مرات باليوم	4-5 مرات باليوم	عالأقل 6مرات باليوم
74	بندورة طازجة أو 2/1 كأس بندورة شيري	قطعة									
75	بندورة مطبوخة يشمل صلصة البندورة أو شوربة بندورة	1/2 كأس									
76	فلقل أحمر	1 فلفل									
77	فلقل أخضر	1 فلفل									
78	خيارأو سلطة خيار	1خيارة									
79	كوسة أوباذنجان (أحط بدائرة الصورة الملائمة)	صورة 37,38,39									
80	بازيلاء,فاصوليا خضراء أو بامية مطبوخة (أحط بدائرة الصورة الملائمة)	صورة 37,38,39									
81	زهرة أو بروكلي	1/2 كأس									
82	بطاطا حلوة (أحط بدائرة الصورة الملائمة)	صورة 28,29,30									
83	ملفوف أخضر أو احمر يشمل الكرنباء	صورة 40,41,42									
84	خس طازج يشمل سلطة الخس (أحط بدائرة الصورة الملائمة)	صورة 40,41,42									
85	جزر,طازج أو مطبوخ, يشمل عصير الجزر	جزرة أو 1/4 كأس عصير									
86	ذرة	كوز									
87	بقدونس,سلري,كوسبرة,بصل أخضر	ملعقة كبيرة من الخضار المفروم									
88	سلطة طازجة من دون صلصة أو زيت (لايشمل ما حددته حتى الان) (أحط بدائرة الصورة الملائمة)	صورة 40,41,42									
89	سلطة طازجة مع صلصة أو زيت (لايشمل ما حددته حتى الان) (أحط بدائرة الصورة الملائمة)	صورة 40,41,42									
90	أفوكادو يشمل سلطة الأفوكادو	ملعقة كبيرة									
91	ليمون	1/2ليمون									
92	بصل	1/4بصلة									
93	ثوم	سن ثوم									
94	شوربة خضار أو أضافة خليط خضار	كأس شوربة									
95	سلطة حمص	ملعقة كبيرة									
96	طحينة	ملعقة كبيرة									
97	سلطة خضار جاهزة الصنع مثل:توركي,مطبوخة,باذنجان,ملفوف	ملعقة كبيرة									
98	خضار مكبوسة ,مخلل لايشمل الزيتون	قطعة									
99	زيتون	10 حبات زيتون									

تحقق أذا تمت الاشارة في كل سطر على الوتيرة-

أذا حجم الوجبة أو الصورة مشار أليها بخط أحط بدائرة حجم

مسلسيات والحلويات

**رقم المنتوج الغذائي**	**وصف الغذاء**	**حجم الوجبة / صورة**	**بأي وتيرة تم أستهلاك المنتوج الغذائي بالمعدل خلال العام الماضي**								
			اقل من مرة في الشهر	1-3مرات في الشهر	1-2 مرات فًي الاسبوع	3-4 مرات فًي الاسبوع	5-6مرات فًي الاسبوع	مرة باليوم	2-3 مرات باليوم	4-5 مرات باليوم	عالأقل 6مرات باليوم
100	شوكولاطة بالحليب أو مرير أو مسلي شوكولاطة من كل نوع	6مكعبات أو 3غرام									
101	مسليات مالحة مثل: بسلي بيجلة,"شكدي مرك"	حفنة أو 25 غرام									
102	كعكة جبنة أو كريما (أحط بدائرة الصورة الملائمة)	<u>صورة 43,44,45</u>									
103	كعك بالخميرة مثل: كوراسون, كوراسون بالشكولاطة,سفجنيوت	قطعة /شريحة									
104	كعكة بسكوت أو تورتيا يشمل كعكة الشكولاطة (أحط بدائرة الصورة الملائمة)	<u>صورة 43,44,45</u>									
105	كعكة فاكهة يشمل كعكة الباي (أحط بدائرة الصورة الملائمة)	<u>صورة 43,44,45</u>									
106	بفل وبسكويت محشي أو مغطى	قطعة									
107	بسكويت عادي يشمل البسكويت الغير مغطى	قطعة									
108	بوظة (كرات او بعيدان)	2 كرات أو حبة									
109	بذر أبيض/ أسود يشمل بذر البطيخ	حفنة أو 25 غرام									
110	جوز,لوز,فستق	حفنة أو 25 غرام									
111	بندق	حفنة أو 25 غرام									
112	زيت المضاف الى السلطة أو الوصفات	ملعقة صغيرة									
113	ميونز يشمل الخفيف	ملعقة صغيرة									
114	عسل ,مربى,شراب فاكهة,شراب الميبل	ملعقة صغيرة									
115	سكر	ملعقة صغيرة									
116	حلويات مصنعة مثل المحلي الخفيف- سوكرزيت	قطعة									

تحقق أذا تمت الاشارة في كل سطر على الوتيرة-

أذا حجم الوجبة أو الصورة مشار أليها بخط أحط بدائرة حجم

<u>المشروبات</u>

**رقم المنتوج الغذائي**	**وصف الغذاء**	**حجم الوجبة / صورة**	**بأي وتيرة تم أستهلاك المنتوج الغذائي بالمعدل خلال العام الماضي**								
			اقل من مرة في الشهر	1-3مرات في الشهر	1-2 مرات فًي الاسبوع	3-4 مرات فًي الاسبوع	5-6مرات فًي الاسبوع	مرة باليوم	2-3 مرات باليوم	4-5 مرات باليوم	عالأقل 6مرات باليوم
117	عصير نكطير يشمل عصير التفاح	كأس									
118	عصير بطعم فاكهة يشمل شراب,سيروب جاهز مخفف	كأس									
119	مشروبات قليلة السعرات الحرارية (الدايت)	كأس									
120	المشروبات الغازية العادية (مع سكر)	كأس									
121	قهوة من دون كفائين	كأس/قدح									
122	قهوة ونسكافه	كأس/قدح									
123	شاي الأعشاب	كأس/قدح									
124	شاي	كأس/قدح									
125	بيرة	1.5 كأس أو علبة									
126	نبيذ حلو,كوكتيل	1 كأس									
127	**مشروبات كحولية حادة**	1 كأس									
